# Effect of protein aggregation in wheat-legume mixed pasta diets on their in vitro digestion kinetics in comparison to “rapid” and “slow” animal proteins

**DOI:** 10.1371/journal.pone.0232425

**Published:** 2020-05-04

**Authors:** Insaf Berrazaga, Claire Bourlieu-Lacanal, Karima Laleg, Julien Jardin, Valérie Briard-Bion, Didier Dupont, Stéphane Walrand, Valérie Micard

**Affiliations:** 1 IATE Agropolymers Engineering and Emerging Technologies, Université Montpellier, CIRAD INRA, Montpellier SupAgro, Montpellier, France; 2 UNH, Unité de Nutrition Humaine, CRNH, Université Clermont Auvergne, INRA, Auvergne, Clermont-Ferrand, France; 3 UMR 1253 STLO Science et Technologie du Lait et de l'Œuf, Agrocampus Ouest, INRA, Rennes, France; 4 Service de Nutrition Clinique, Centre Hospitalier Universitaire (CHU) Gabriel Montpied, Clermont-Ferrand, France; Consejo Superior de Investigaciones Cientificas, SPAIN

## Abstract

The aim of this work was to evaluate the impact of incorporating different legume flours (faba bean, lentil or split pea flours) on the pasta protein network and its repercussion on in vitro protein digestibility, in comparison with reference dairy proteins. Kinetics and yields of protein hydrolysis in legume enriched pasta and, for the first time, the peptidomes generated by the pasta at the end of the in vitro gastric and intestinal phases of digestion are presented. Three isoproteic (21%) legume enriched pasta with balanced essential amino acids, were made from wheat semolina and 62% to 79% of legume flours (faba bean or F-pasta; lentil or L-pasta and split pea or P-pasta). Pasta were prepared following the conventional pastification steps (hydration, mixing, extrusion, drying, cooking). Amino acid composition and protein network structure of the pasta were determined along with their culinary and rheological properties and residual trypsin inhibitor activity (3–5% of the activity initially present in raw legume flour). F- and L-pasta had contrasted firmness and proportion of covalently linked proteins. F-pasta had a generally weaker protein network and matrix structure, however far from the weakly linked soluble milk proteins (SMP) and casein proteins, which in addition contained no antitrypsin inhibitors and more theoretical cleavage sites for digestive enzymes. The differences in protein network reticulation between the different pasta and between pasta and dairy proteins were in agreement in each kinetic phase with the yield of the in vitro protein hydrolysis, which reached 84% for SMP, and 66% for casein at the end of intestinal phase, versus 50% for L- and P-pasta and 58% for F-pasta. The peptidome of legume enriched pasta is described for the first time and compared with the peptidome of dairy proteins for each phase of digestion. The gastric and intestinal phases were important stages of peptide differentiation between legumes and wheat. However, peptidome analysis revealed no difference in wheat-derived peptides in the three pasta diets regardless of the digestion phase, indicating that there was a low covalent interaction between wheat gluten and legume proteins.

## Introduction

Diversifying protein sources in food intake, particularly increasing the consumption of vegetable proteins could reduce the risks to health and to the environment associated with the production and the excessive consumption of animal proteins in Western countries [1,2]. However alone, each vegetable protein, may lack certain essential amino acids such as sulfur amino acid, threonine or/and lysine [3]. Blending two complementary vegetable proteins, such as cereals deficient in lysine and threonine [4], and legumes deficient in sulfur amino acids [5], could result in balanced essential amino acid composition [6,7]. In addition, cereals and legumes present proteins with contrasted biochemical properties. Wheat proteins are mainly composed of glutenins soluble in acid or alkali solutions, and gliadins, which are soluble in hydro-alcoholic solutions. These proteins form inter- and intra-molecular disulfide bonds during food processing, leading to the formation of a three dimensional gluten network [8]. Globulins, which are soluble in saline solution, and albumins, which are soluble in water are minor proteins in wheat [9]. Conversely, globulins represent 50% to 80% and albumins 10% to 35% of legume proteins, along with minor proteins like prolamines and glutelins [10–12]. Enzymes, enzyme-inhibitors and lectins, all involved in seed defense mechanisms, are also present in legumes and are anti-nutritional factors in the human diet. Depending on their sedimentation coefficient, legume proteins are classified as legumin (globulin 11S), vicilin and convicilin (globulin 7S) and albumin (2S).

Several authors have studied the supplementation of wheat pasta with legume flours (lupin, lentil, chickpea, pea, faba bean and black gram) with substitution rates ranging from 5% to 100% [8,13–23]. These studies mainly evaluated the chemical composition and protein network structure of pasta and their culinary, rheological and sensory properties. They showed that adding legume to pasta impaired their culinary and rheological properties by increasing cooking loss and reducing firmness, and their overall sensory acceptability. These negative impacts varied with the nature of the legume and the level of incorporation. In faba bean enriched pasta, it was demonstrated that these changes in culinary and rheological properties were linked to weakening of the protein network structure with an increasing legume:wheat ratio. A linear decrease in the covalent bonds at the expense of weak protein bonds was observed in legume enriched pasta [22]. The incorporation of 0% to 100% faba bean led therefore to a 30% decrease in covalent protein bonds in pasta [22,24].

The changes in the structure of the protein network in wheat pasta when enriched with faba bean flour also affected in vitro protein digestibility [22,24,25]. However, the effect of the type of legume flour used to enrich pasta on the pasta protein network structure and digestibility is not yet well understood. Neither the protein structure nor its digestibility have been compared to the digestive behavior of animal proteins.

The main objective of this work was thus to evaluate the structural modifications of the protein network induced by the incorporation of three different legume flours i.e. faba bean, lentil or split pea flour in wheat pasta and their repercussions on in vitro pasta protein digestibility. All the pasta were enriched with legume flour to reach 21% protein content (instead of 13% in 100% wheat pasta) and their essential amino acid composition to meet the requirements for healthy adult subjects [26]. In addition, the influence of the nature of the legume on trypsin inhibitory activity and on the culinary and rheological characteristics of the pasta were assessed. Pasta protein digestibility kinetics were compared with reference dairy proteins known to be digested at different rates in human (casein and soluble milk proteins, slow and rapid proteins, respectively) [27]. For the first time, the peptides generated during the in vitro gastric and intestinal phases of digestion of the three cereal-legume pasta diets were analyzed and compared with the peptidome generated during the digestion of casein and the soluble milk proteins.

## Materials and methods

### Raw materials

Faba bean (*Vicia faba*), green lentil (*Lens culinaris*) and green split pea (*Pisum sativum L*.) flours were provided by GEMEF industries (Aix-en-Provence, France), Celnat (Saint Germain Laprade, France) and Moulin des Moines (Krautwiller, France), respectively. Wheat (*Triticum durum*) semolina was supplied by La Semoulerie de Bellevue (Groupe Panzani, Marseille, France). Reference animal proteins, casein and soluble milk proteins (SMP), in the form of sodium caseinate and Prolacta®, respectively, were provided by Lactalis (Torcé, France). This study was carried out upstream of an in vivo study conducted using old sarcopenic rats to evaluate the in vivo protein digestibility and retention of legume enriched pasta. Cow’s milk proteins were used as a reference given their high biological value and their good digestibility i.e. ≥ 96% [28,29] and utilization in the body. These animal proteins were mixed with carbohydrate and lipids, to form the casein and soluble milk protein diets designed for the in vivo study. Casein and soluble milk protein diets containing 19% db of proteins, 70% db of carbohydrates (starch and cellulose) and 7% db of lipids were compared to legume enriched pasta diets.

### Pasta production

The pasta were produced at the IATE joint research unit (SupAgro-INRA-UM-CIRAD, Montpellier, France). Hydration, mixing and extrusion (40°C) were carried out in a pilot-scale pasta extruder (Bassano, Lyon, France) according to the WO2016097328 A1 patent [30]. Pasta were dried at a low temperature (55°C, 15 h) in a pilot drier (AFREM, Lyon, France).

Different proportions of the three legume flours were mixed each with durum wheat semolina to obtain three legume-wheat mixed pasta all enriched in protein up to 21% (versus 13% for a standard durum wheat pasta). Depending on the protein content of the raw legume flours, the three isoprotein legume-wheat mixed pasta were produced as follows:

- Faba bean-wheat pasta (F-pasta): Pasta with 38% wheat semolina and 62% faba bean flour;
- Green lentil-wheat pasta (L-pasta): Pasta with 35% wheat semolina and 65% lentil flour;
- Green split pea flour-wheat pasta (P-pasta): Pasta with 21% wheat semolina and 79% split pea flour.

Dried pasta were cooked for their respective optimal cooking times + 1 minute and re-dried at low temperature (40°C, 24 h) in order to avoid protein reactions, and then ground. In the rest of this paper, this material is referred to as ‘legume enriched pasta diet’. It was used in this form for SE-HPLC, trypsin inhibitory activity and in vitro digestibility experiments.

### Cooking and rheological properties of pasta

Dried pasta were cooked in demineralized boiling water (100°C) containing 0.7% (w/v) of sodium chloride for their own optimal cooked time (OCT) + 1 minute to allow complete starch gelatinization according to the AACC approved method (66–50). Cooking losses were measured in triplicate according to the protocol described in Petitot et al. [8]. The rheological properties of the cooked pasta were analyzed using a TA-XTplus (Stable Micro Systems, Scarsdale, USA) texture profile analyzer. Texture profile analysis (TPA) was carried out to determine pasta firmness as described in Petitot et al. [31]. Five replicates of three different cooking steps (n = 15) were performed for each pasta.

### Chemical composition of pasta

The Kjeldahl procedure (NF V 03–050, 1970) was used to determine total protein content using a conversion factor of 6.25 for legume flours and 5.70 for wheat semolina. An enzymatic assay kit (Megazyme, Co. Wicklow, Ireland; AACC method 76–13.01) was used to determine total starch content. Amino acid profiles, lipid content and soluble and insoluble fibers were determined by Agrobio (Rennes, France) according to the CEE 152/2009 (2009), decree 08-09-1977, AOAC 991–42 and AOAC 993–19 methods, respectively.

### Trypsin inhibitory activity of legume enriched pasta diets

Trypsin inhibitory activity (TIA) was analyzed in triplicate on raw legume flours, wheat semolina, and on each cooked (at OCT + 1 minute) and re-dried pasta according to the standardized method ISO 14902 (2009).

### SE-HPLC analysis of proteins in legume enriched pasta and animal diets

Two successive protein extractions were performed in triplicate as described by Petitot et al. [31] on legume enriched pasta and animal diets. The first extraction in sodium dodecyl sulfate (SDS) was used to extract weakly linked proteins (linked by electrostatic, hydrophobic and hydrophilic bonds), also called “SDS-soluble” proteins. In order to ensure disruption of the disulfide bonds, a second extraction was performed in SDS-dithioerythritol (DTE) with a sonification step (called “SDS+DTE-soluble” proteins, linked by S-S bonds). The remaining protein fraction, insoluble in SDS and DTE (hereafter “non-extractable proteins”), represented the proteins linked by covalent bonds other than disulfides. The protein size distribution of SDS-soluble and SDS+DTE-soluble extracts was analyzed by size-exclusion high performance liquid chromatography (SE-HPLC) according to Morel et al. [32] and as described in Petitot et al. [31]. The results are reported relative to the total extractable proteins in SDS and DTE of raw materials i.e. blends of wheat semolina and F- or L- or P-flours for F- or L- or P-pasta diets, respectively, and of casein and soluble milk protein diets for animal proteins.

### In vitro protein digestion kinetics of legume enriched pasta and animal diets

#### In vitro digestion

In vitro protein digestion was performed in a stirred water bath at 37°C according to Minekus et al. [33]. The enzyme activities of salivary amylase, pepsin and pancreatin were measured in triplicate as defined in the protocol. Briefly, simulated digestion consisted in an initial two-minutes “oral phase” including human salivary amylase (330 U/mg of amylase, A0521, Sigma, St. Louis, US, 75 U/mL final digestion mixture), followed by a 60-minutes “gastric phase” including porcine pepsin (122 U/mg of pepsin, P7125, Sigma, St. Louis, US, 2190 U/mL final digestion mixture) and a 120-minutes “intestinal phase” using porcine pancreatic extract and porcine bile (413.2 USP/mL of pancreatin, P7545, 7.9 U trypsin/mg and added at 100 U/mL trypsin in the final digestion mixture and 3.9 mmol/g of bile, B8631 both from Sigma, St. Louis, US). The in vitro protein digestion was carried out in triplicate for each legume enriched pasta diet or animal diet. Kinetics were measured at 0, 2, 4, 17, 32, 62, 64, 72, 92, 122, 182 min. Protein hydrolysis was stopped by adding 50 μl/mL of mixture of pefabloc 0.1 M (76307, Sigma, St. Louis, US) and the samples were immediately deep frozen in liquid nitrogen.

#### Determination of the degree of protein hydrolysis

After centrifugation (4000 g, 20 min, 4°C) to determine the free amino group content of the digestate, the degree of protein hydrolysis was evaluated with a ninhydrin assay performed according to Prochazkova et al. [34]. The degree of protein hydrolysis (°DH) based on the amount of free amino group released at the beginning of digestion (t0), during digestion (tx) and after total hydrolysis with HCl 6N for 24 h at 105°C (ttot) was determined and calculated according to the Eq (1):

$${}^{\circ}DH\;(\%)=\frac{{\left[NH2\right]}_{\left(tx\right)}-{\left[NH2\right]}_{\left(t0\right)}}{{\left[NH2\right]}_{\left(ttot\right)}-{\left[NH2\right]}_{\left(t0\right)}}\times 100$$ (1)

#### SDS Page protein profile during in vitro digestion of the legume enriched pasta and animal protein diets

Electrophoretic analysis were performed using 4–20% polyacrylamide TruPAGE® 12-well precast gels (Sigma-Aldrich, Saint Quentin Fallavier, France) run for 60 min at 50 mA per gel in MES-SDS running buffer (Sigma-Aldrich, Saint Quentin Fallavier, France). After centrifugation at 4000 g for 20 min, SDS Page was performed in reducing conditions on the supernatants of undigested initial sample (T0), gastric digestate (60 min of gastric digestion) and intestinal digestate (120 min of intestinal digestion). Wheat semolina, legume flours, legume enriched pasta diets, casein and soluble milk protein diets were suspended in simulated salivary fluid [33] without enzymes and considered as undigested initial samples (T0). For all samples, except gastric digestate, 30 μL of the supernatant was diluted in a final volume of 300 μL containing 3 μL of DTT (5 mM final concentration), 75 μL of TruPAGE LDS sample buffer (4X) and 192 μL distilled water. For gastric digestate, a higher concentration of the supernatant was needed to better visualize protein bands. Gastric digestate supernatants were diluted with TruPAGE LDS sample buffer (4X) containing DTT at a final concentration of 5 mM at a volume ratio digestate-to-sample buffer of 1:3 (v/v). Samples were inerted with argon and heated at 70°C for 10 min. For the determination of the polypeptide profiles of wheat semolina and legume flours, 20 μL were loaded into adjacent wells in the gel. For F-, L- and P-pasta diets, 20 μL of each undigested or gastric samples and 30 μL of intestinal samples were loaded into adjacent wells in the gel. For caseins and soluble milk protein diets, 7 μL of each undigested or gastric samples and 10 μL of intestinal samples were loaded into adjacent wells in the gel. Mark 12 Unstained Standard (Invitrogen, Thermo Fisher scientific Waltham, USA) was used as a molecular weight (MW) marker. Blank samples containing only enzymes (human salivary alpha amylase, porcine pepsin and pancreatin) were deposited on control gels to visualize their SDS Page prints at the concentrations present in the digestive fluids. Gels were fixed in 30% (v/v) ethanol, 10% (v/v) acetic acid and 60% (v/v) deionized water for at least one hour and were rinsed three times in deionized water before staining with Bio-Safe Coomassie Staining Solution (Bio-rad, US). Image analysis of SDS Page gels was carried out using GelAnalyzer software (2010, http://www.gelanalyzer.com). The MW bands of F-, L- and P-pasta diets were attributed according to SDS Page of the undigested raw matters (wheat semolina, F-, L- and P-flours) used for pasta production (S1 Fig). For undigested raw matter i.e. legume flours (S1 Fig), polypeptides of molecular weights around 18, 21.5 and 32 to 46 kDa could be attributed to basic and acidic legumin subunits, respectively, according to previous studies [12,35,36]. Polypeptides of 12, 14, 16, 29, 50 and 66 to 82 kDa could be ascribed to the polypeptide constituents of vicilin and convicilin, respectively [35,37,38]. Polypeptides detected around 24–25 and 3.5 to 6 kDa were assigned to albumins [39,40]. For wheat semolina (S1 Fig), gliadin polypeptides, low MW and high MW glutenins could be observed at 30–80 kDa, 30–55 kDa and 100–140 kDa, respectively [41]. Bands with MW below 30 kDa could be attributed to albumins provided from wheat semolina [41]. For milk proteins i.e. casein and SMP, MW of polypeptides were identified according to Berrazaga et al. [38]. The polypeptide band intensity was determined by densitometry using the 1D gel electrophoresis image analysis software Gel Analyzer 2010a. Densitometry analysis of the SDS Page gels was used to evaluate polypeptide composition of the legume flours, wheat semolina, legume enriched pasta diets, casein and soluble milk protein diets. In each gel, the intensity of each band in each line was divided by the sum of total band intensities of the line to determine the relative content of each peptide or polypeptide in a sample in comparison to the extractable proteins in the digestive fluids.

#### Peptidome analysis by tandem mass spectrometry

*Identification of peptides*. Mass spectrometry (MS) analysis was conducted as described previously [42]. Briefly, a nano-RSLC Dionex U3000 system fitted to a Q-Exactive mass spectrometer (Thermo Scientific, San Jose, USA) equipped with a nanoelectrospray ion source was used. Digestate samples (in duplicate) were normalized according to their protein content (X μg/mL), diluted 100 times in the injection buffer and filtered (0.45 μm cut-off) before concentration on a μ-precolumn pepMap100 (C18 column, 300 μm i.d. × 5 mm length, 5 μm particle size, 100 Å pore size; Dionex, Amsterdam, The Netherlands) and separation on a PepMap RSLC column (C18 column, 75 μm i.d. x 150 mm length, 3 μm particle size, 100 Å pore size; Dionex).

Peptides were separated at a flow rate of 0.3 μL.min-1 using solvent A [2% (v/v) acetonitrile, 0.08% (v/v) formic acid and 0.01% (v/v) TFA in HPLC gradient grade water] and solvent B [95% (v/v) acetonitrile, 0.08% (v/v) formic acid and 0.01% (v/v) TFA in HPLC gradient grade water]. The elution gradient was first from 5% to 35% solvent B over a period of 87 min, then increased to 85% solvent B over 3 min before column re-equilibration. The mass spectra were recorded in positive mode using the m/z range 250–2000. The resolution of the mass analyzer for m/z of 200 amu (atomic mass unit) was set in the acquisition method to 70 000 for MS and to 17 500 for MS/MS. For each MS scan, the 10 most intense ions were selected for MS/MS fragmentation and excluded from fragmentation for 15 s.

Peptides were identified from the MS/MS spectra using X!Tandem Pipeline software (https://pubs.acs.org/doi/abs/10.1021/acs.jproteome.6b00632) against protein databases downloaded from www.ncbi.nlm.nih.gov (NCBI:txid3906–875 protein sequences from *Vicia faba*, NCBI:txid3888–7,240 proteins from *Pisum sativum*, NCBI:txid3864–394 proteins from *Lens culinaris*, NCBI:txid4567–3,211 proteins from *Triticum turgidum subsp*. *durum*) and uniprotkb.org (taxon identifier 3904–1,410 protein sequences from *Vicia*) to which the common Repository of Adventitious Protein (http://thegpm.org/crap) was added. The possible post-translational modifications allowed were serine or threonine phosphorylation and methionine oxidation. Peptides identified with an e-value < 0.05 were automatically validated. The peptide false discovery rate obtained with these parameters was less than 0.1%.

*Quantification of peptides*. Each identified peptide was quantified using MassChroQ software [43]. The alignment method used common identified peptides between two LC-MS runs as landmarks, which were then used to compute a deviation curve using a linear interpolation. This label-free MS-based peptide quantification enabled semi-quantitative non-differential single class analysis of the peptides. A m/z width of 10 ppm was used to perform extracted ion chromatograms (XIC) of peptides in time-aligned chromatograms, and the area under the curve (AUC) was then quantified. When a peptide was measured with several charge states, all ion intensities were summed.

Statistical analysis was performed using R software, version 3.3.1. The abundance of each peptide (area under the curve), determined at three time points (at T0, at the end of the gastric phase, and at the end of the intestinal phase), of two digestions was log10-transformed and the maximum abundance was set to 1. These values were used for the ascending hierarchical clustering (hclus function, stats package) of the 2,447 peptides resulting from hydrolysis of the pasta diets based on the minimum within-cluster variance using Ward’s agglomeration. The heatmap.2 function was used to display the heatmap and its dendrogram. Clusters were characterized (catdes function, FactoMiner package) using the biochemical characteristics. A chi-square test was performed for qualitative variables (protein origin), and a one-way analysis of variance for quantitative variables (peptide abundance, leucine and glutamine content). V-tests indicated whether the modality category was significantly overrepresented (v > 2) or underrepresented (v < -2). The statistical significance level was p < 0.05. The number of unique peptide sequences found in duplicate injection of digestate samples was called n_u_.

### Statistical analysis

Results are expressed as means ± SD. Variance analysis (ANOVA) and Fisher’s post-hoc test (protected least significant difference) were used to determine significant differences between means. Differences were considered significant with a 5% risk of error. Means with different letters are significantly different at p < 0.05. Statistical analysis of data was performed using the StatView® software (SAS, Inc. Institute, Release 5, 1992–82) and R software, version 3.3.1 for peptidome analysis.

## Results

### Chemical composition of legume enriched pasta diets

The composition of faba bean, lentil and split pea enriched pasta diets is given in Table 1.

**Table 1:** Chemical composition and amino acid composition of pasta and animal protein diets.

Components		Casein	SMP	F-pasta	L-pasta	P-pasta
Total starch (%, db)		63.2	63.2	64.4	59.1	58.5
LS (% of total starch)				53.2	54.5	71.6
WS (% of total starch)				46.8	45.5	28.4
Total fibers (%, db)		6.8	6.8	7.4	11.7	10.4
Soluble fibers (% of total fibers)				18.9	17.9	19.2
Insoluble fibers (% of total fibers)				81.1	82.1	80.8
Total lipids (%, db)		7.0	7.0	1.4	1.6	2.6
Total proteins (%, db)		19.0	19.0	21.2	21.2	21.5
LP (% of total protein)				77.4	79.1	87.5
WP (% of total protein)				22.6	20.9	12.5
		Casein	SMP^2^	F-pasta	L-pasta	P-pasta
Essentiel amino acids (mg/g protein)	WHO/FAO/UNU^1^ recommendation					
Histidine	15	26.1	20.0	26.9	25.3	25.1
Isoleucine	30	48.5	50.5	43.1	45.2	45.5
Leucine	59	87.7	120.3	79.1	80.3	78.9
Lysine	45	74.9	96.5	54.0	57.2	66.9
Methionine + cysteine	22	45.7	51.4	26.3	26.1	26.0
Cysteine		14.3	30.7	13.9	15.8	13.5
Phenylalanine + tyrosine	30	88.2	72.2	74.1	81.4	83.4
Tyrosine		40.4	34.3	26.1	28.7	29.0
Threonine	26	39.5	50.5	37.0	37.2	38.3
Tryptophan	6	10.6	20.7	9.0	7.5	7.1
Valine	39	65.9	51.4	48.2	49.7	49.5

^1^WHO/FAO/UNU [26] pattern for adults

^2^ Amino acid composition of SMP was calculated from [44]

F-pasta = faba bean enriched pasta diet; L-pasta = Lentil enriched pasta diet; P-pasta = split pea enriched pasta diet; SMP = Soluble milk protein diet; casein: casein diet; LS = Starch from legume; WS = Starch from wheat semolina; LP = Protein from legume flour; WP = Protein from wheat semolina

All legume enriched pasta diets were formulated in order to reach an isoprotein content of 21% db. Depending on the initial amount of proteins in legume flour, 77%, 79% and 87% of the total pasta diet protein contents were faba bean (F-pasta diet), lentil (L-pasta diet) and split pea (P-pasta diet) proteins, respectively, versus 23%, 21% and 13% of wheat proteins. Minor variations in non-protein components resulted from the legume isoprotein enrichment: the F-pasta diet contained ~5% more total starch and, ~3% less total fibers (insoluble fibers) than the L- and P-pasta diets. The L- and P-pasta diets had very similar total starch and fiber compositions.

Amino acid composition of legume enriched pasta diets and casein and soluble milk protein diets is given in Table 1. They are all above the WHO/FAO/UNU recommended levels [26]. Overall, the essential amino acid composition of the three legume enriched pasta diets was similar, the main difference being higher lysine content in the P-pasta diet than in the L- and F-pasta diets, linked to the lower lysine content in wheat proteins. Cysteine content, likely involved in S-S bridging, was 14–17% higher in the L-pasta diet than in the F- and P-pasta diets. Aromatic amino acid contents (phenylalanine, tyrosine which can form di-tyrosine products) were 10–12.5% higher in the L- and P-pasta diets than in the F-pasta diet. In comparison to pasta diets, casein and SMP diets were richer in most amino acids except for cysteine, which was equivalent in the casein diet, and phenylalanine, which was present in lower amounts in SMP. Sulfur amino acid contents were twice as high in animal protein diets, particularly in the SMP diet, which was 2-fold richer in cysteine. Much higher lysine concentration were also found in milk protein diets, especially in SMP diet, whereas tyrosine was more concentrated in the casein diet.

### Protein aggregation in the protein network of legume enriched pasta diets compared with animal protein diets

**Fig 1:**
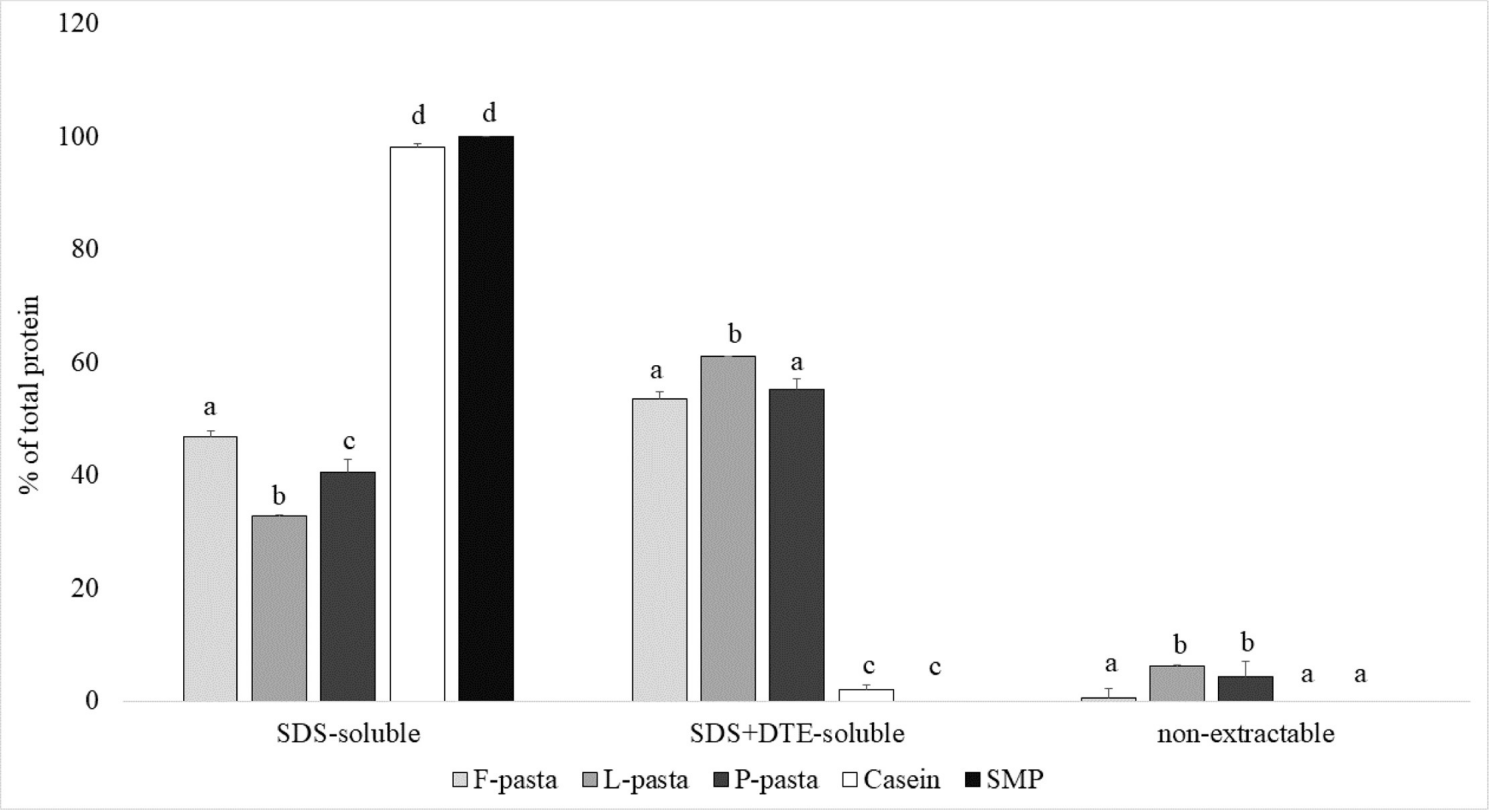
Protein aggregation of legume enriched pasta diets by SE-HPLC in comparison with animal protein diets. Different letters represent significant difference between groups (p < 0.05). F-pasta = faba bean enriched pasta diet; L-pasta = Lentil enriched pasta diet; P-pasta = split pea enriched pasta diet; SMP = Soluble milk protein diet; Casein = casein diet.

Results of SE-HPLC analysis of legume enriched pasta diets and milk protein diets are presented in Fig 1. F-, L- and P-pasta diet proteins were 41% to 47% soluble in SDS. Their solubility thus strongly decreased compared to the initial legume flours (SDS-soluble = 97%, S2 Fig) and milk protein diets (98–100%). This indicates a smaller amount of weakly bonded proteins in processed pasta diets than in milk protein diets to the benefit of S-S linked proteins and even a small amount of proteins linked by other covalent bridges (Fig 1). Only a slight difference was observed between the three legume enriched pasta diets, with a 1.2 to 1.4 fold higher amount of proteins soluble in SDS in the F-pasta diet. Conversely, the proportion of SDS+DTE soluble proteins was higher in the L-pasta diet than in the P- and F-pasta diets. This difference points to more S-S bridges in the L-Pasta, probably linked to its higher cysteine content. Indeed, cysteine content was 14% higher in the L-pasta diet than in the F-pasta diet and 17% higher than in the P-pasta diets (Table 1). The L- and P-pasta diets also contained 6.2% and 4.4% of non-extractable proteins, respectively. These proteins could be linked by isopeptide bonds [22] as dityrosine bridges, as their tyrosine content was respectively +10 and 11% higher (Table 1) than in the F-pasta diet.

### Cooking and rheological properties of pasta

The cooking and rheological properties of pasta are listed in Table 2.

**Table 2:** Cooking and rheological properties of pasta.

	F-pasta	L-pasta	P-pasta
Optimal cooking time (min)	8.7^a^	8.5^b^	9.0^c^
Cooking loss (%, db)	10.2 ± 0.4^a^	9.2 ± 0.6^a^	12.3 ± 0.6^b^
Firmness (N)	3.48 ± 0.29^a^	5.29 ± 0.47^b^	4.03 ± 0.35^c^

Means with different superscripts in the same row are significantly different at p < 0.05

F-pasta = faba bean enriched pasta; L-pasta = lentil enriched pasta; P-pasta = split pea enriched pasta

Optimal cooking time (OCT) and firmness and, to a lesser extent, cooking losses were significantly affected by the nature of legume used to enrich the pasta, in agreement with Laleg et al. [21]. The differences between protein network structures according to SE-HPLC results could explain the differences in pasta cooking properties. Indeed, L-pasta with the highest covalently reticulated protein network (9 to 13% more covalently linked proteins) were also the firmest pasta and also had low cooking losses. Conversely, F-pasta with the weakest protein network structure had the lowest firmness. P-pasta, whose protein network aggregation closely resembled that of F-pasta, notably in terms of covalently linked protein fraction, required a longer cooking time due to its higher proportion of salt soluble globulin provided by legume flour. The larger proportion of salt soluble globulin also very likely resulted in higher particle losses in the cooking water [13,15]. More generally, the lack of disulfide bonded proteins in P- and F-pasta, evidenced by SE-HPLC analysis, could be responsible for increased loss of particles in the cooking water [24].

### Trypsin inhibitory activity in legume enriched pasta diets

Trypsin inhibitory activity (TIA) of legume flours and legume enriched pasta diets is given in Table 3.

**Table 3:** Trypsin inhibitory activity of legume flours and legume enriched pasta diets.

	TIA (mg/g db)	
Products	Flours	Pasta^1^
Faba bean	11.29 ± 0.14^a^	0.33 ± 0.0^a^
Lentil	9.37 ± 0.14^b^	0.40 ± 0.01^b^
Pea	7.78 ± 0.08^c^	0.38 ± 0.0^c^

Means with different superscripts in the same column are significantly different at p < 0.05

^1^Faba bean enriched pasta, lentil enriched pasta and split pea enriched pasta diets

The TIA of legume flours were in the first third of the intervals reported in the literature for such flours, ranging from 1–29 mg/g [21,45–50]. Pasta processing, i.e. drying at 55°C, but more probably the cooking step, drastically reduced TIA, which remained less than 5% of the initial TIA content. Complete degradation was obtained by Zhao et al. [14] by drying pasta enriched with 20% pea or lentil flour at 70°C. Laleg et al. [21] dried pasta at 55°C, like in our study, then cooked and freeze dried 100% legume pasta and obtained 18–32% residual TIA.

### In vitro protein digestion kinetics of legume enriched pasta diets compared with animal protein diets

**Fig 2:**
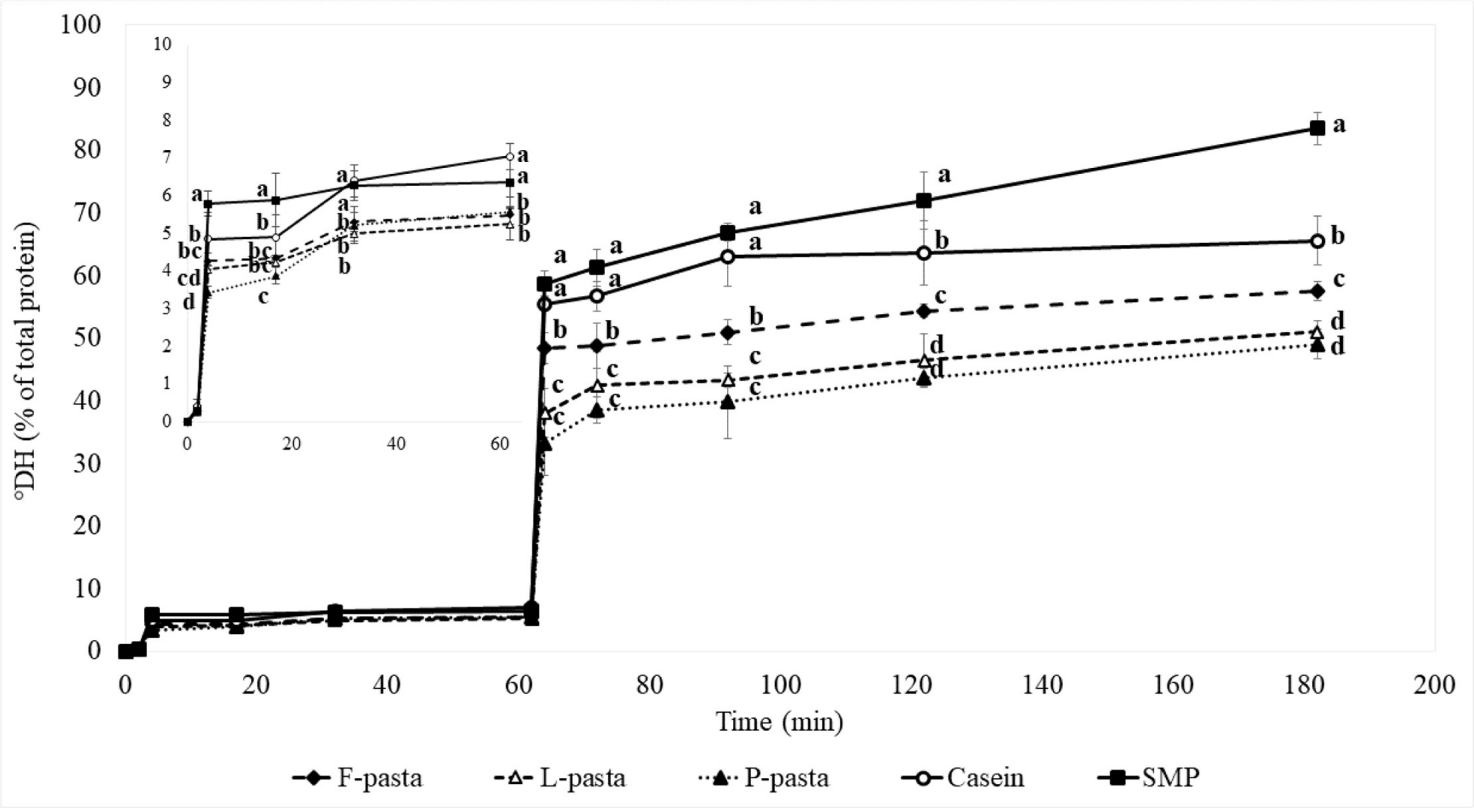
Kinetics of in vitro protein digestion of legume enriched pasta and animal protein diets. Significant differences are indicated per time point (p < 0.05). Different letters represent significant difference between groups (p < 0.05). Oral and gastric protein digestions from 0 to 62 minutes are also presented in the enlarged figure. F-pasta = faba bean enriched pasta diet; L-pasta = lentil enriched pasta diet; P-pasta = split pea enriched pasta diet; SMP = soluble milk protein diet; Casein = casein diet.

Kinetics of the three oral, gastric, intestinal phases of vitro protein digestion of the legume enriched pasta i.e. F-, L- and P-pasta diets, and milk protein diets i.e. casein and SMP diets are presented in Fig 2. At the end of the oral phase, the degree of proteolysis approached zero (about 0.3%) and did not differ statistically between samples. After the gastric and intestinal phases, the degree of proteolysis increased to reach 5–7% and 50–84% of total proteins, respectively. At the end of the intestinal phase, casein and SMP diets were about 18 and 40% more digested than legume enriched pasta diets, respectively.

Focusing now on digestion kinetics, the gastric phase was characterized by slow rates of peptide release whatever the diets. On the contrary, in all the diets, the intestinal phase was characterized by a rapid rate of hydrolysis in the first two minutes, to reach a hydrolysis degree of 55% and 59% for the casein and SMP diets, respectively versus 48%, 38% and 33% for the F-, P- and L-pasta diets, respectively. After this initial period, all the kinetics slowed down to reach an intestinal hydrolysis degree of ~50% for the L- and P-pasta diets, 58% for the F-pasta diet, ~66% for the casein diet and over 80% for the SMP diet.

### SDS Page of legume enriched pasta diets and animal protein diets after gastric and intestinal hydrolysis

**Fig 3:**
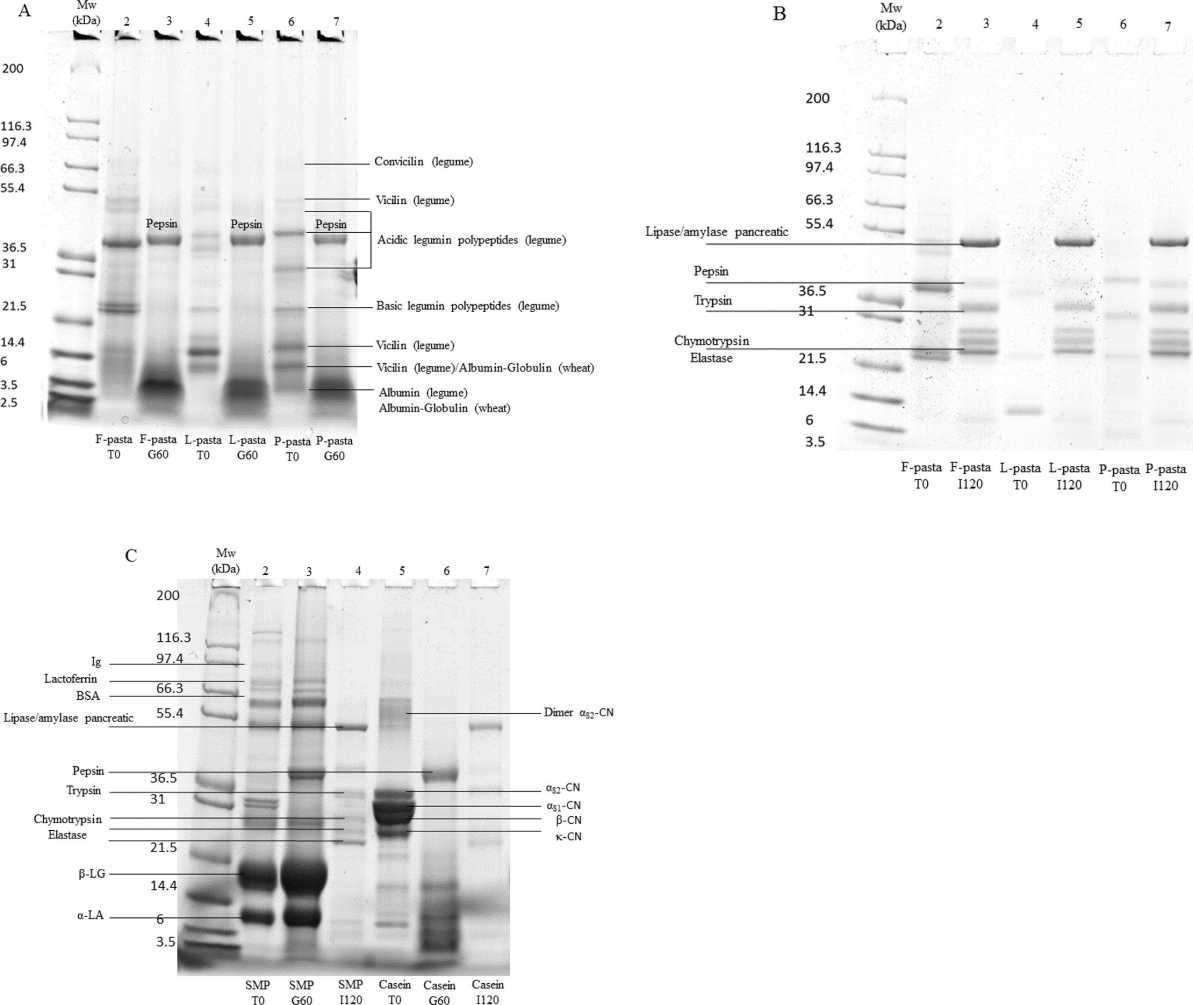
Electrophoretic patterns (T = 12%) under reducing conditions of undigested pasta diets T0 (lanes 2, 4 and 6, Fig 3A) and digested pasta diets G60 after 60 minutes of gastric digestion (lanes 3, 5 and 7, Fig 3A), of digested pasta diets I120 after 120 minutes of intestinal digestion (lanes 3, 5 and 7, Fig 3B), of undigested soluble milk protein and casein diets (lanes 2 and 5, Fig 3C), digested soluble milk protein and casein diets G60 after 60 minutes of gastric digestion (lanes 3 and 6, Fig 3C) and of digested soluble milk protein and casein diets I120 after 120 minutes of intestinal digestion (lanes 4 and 7, Fig 3C). In the lanes, MW = molecular weight markers; F-pasta = faba bean enriched pasta diet; L-pasta = lentil enriched pasta diet; P-pasta = split pea enriched pasta diet; C = Casein diet; SMP = Soluble milk protein diet; α_S2-_casein dimers; α_S1-2-_, β- and κ-CN = casein monomers; β-LG = β-lactoglobulin; α-LA = α-lactalbumin; Ig = immunoglobulin; BSA = bovine serum albumin.

Using SDS Page under reducing conditions, we evaluated the polypeptide profiles of supernatants of reduced T0, gastric (G60) and intestinal (I120) times of both controls (samples without digestive enzyme or undigested samples) and digestates (Fig 3).

The electrophoretic patterns of undigested F-, L- and P-pasta diets (T0, Fig 3A, lines 2, 4, 6) presented bands attributed to vicilin (50, 16 and 14.5 kDa) representing 12% to 37% of total polypeptides in legume enriched pasta diets according to densitometry analysis (result not shown), acidic legumin subunits (at 32 and 46 kDa; 17% to 23% of total polypeptides) and basic legumin subunits (18 and 21.5 kDa; 17% to 24% of total polypeptides). F- and P-pasta diets also presented lower molecular weight proteins or peptides (3.5 to 6 kDa). In the undigested milk protein diets, the casein protein diet (Fig 3C, lane 5) was visible in the form of four adjacent bands corresponding respectively to alpha-s2 (34 kDa), alpha-s1 (31 kDa), beta (28 kDa), and kappa caseins (25 kDa) and representing 11, 15, 13 and 12% of total polypeptides, respectively. The undigested SMP diet (Fig 3C, lane 2) was characterized by more than 10 spots spread from high molecular weight (MW) (around 116 kDa), intermediate MW (45 to 66 kDa) and low MW (6 to 31 kDa). The main bands were α-lactalbumin (14 kDa, 19% of total polypeptides), β-lactoglobulin (18 kDa, 34% of total polypeptides), bovine serum albumin (66 kDa, 2% of total polypeptides), lactoferrin (77 kDa, 3% of total polypeptides) and immunoglobulin (97 and 116 kDa, 4% of total polypeptides).

Considering the end of the gastric phase (G60) (Fig 3A and 3C), a 38 kDa band corresponding to pepsin or human salivary amylase was observed in all the polypeptide profiles compared to our “blank enzyme” (result not shown). In the legume enriched pasta diet polypeptide profiles (Fig 3A, lanes 3, 5 and 7), all the bands present in the undigested samples were hydrolyzed, generating peptides of low molecular weight (2.5 to 10 kDa) regardless of the type of legume. A higher proportion of these peptides was detected in the F-pasta diet. The main casein subunits were also hydrolyzed and generated peptides of low molecular weight (3 to 14 kDa) (Fig 3C, lane 6). In the SMP diet (Fig 3C, lane 3), the three fine bands corresponding to residual caseins and two bands of higher molecular weight (97.4 and 130 kDa), observed initially in undigested sample at T0, were hydrolyzed after 60 minutes of gastric digestion. However, most of the other SMP bands persisted, notably those corresponding to β-lactoglobulin and α-lactalbumin (18 kDa and 14 kDa).

Considering the end of the intestinal phase (I120) polypeptide profiles (Fig 3B and 3C), we observed bands corresponding to digestive enzymes i.e. 51 kDa (porcine pancreatic lipase) and/or porcine pancreatic alpha amylase; 38 kDa (pepsin or human salivary amylase) and 34 kDa (porcine pancreatic enzymes i.e. trypsin), 24–28 kDa (chymotrypsin or elastase), as previously observed by Cozzone et al. [51] and by Sanchón et al. [52]. Two minor bands were also detected at 14.4 and 6 kDa that could be pancreatic enzymes as they were also observed in the blank pancreatic sample (result not shown). At the end of the intestinal phase, i.e. after 182 minutes of digestion, in all the matrices, most of the bands from initial (undigested) samples were hydrolyzed into small peptides (less than 2.5 kDa) which, except for a very small amount of 8 kDa peptides, were not detected by SDS Page. To go further, peptidome analysis of the samples was undertaken.

### Peptidome of legume enriched pasta diets and animal protein diets after gastric and intestinal hydrolysis

**Fig 4:**
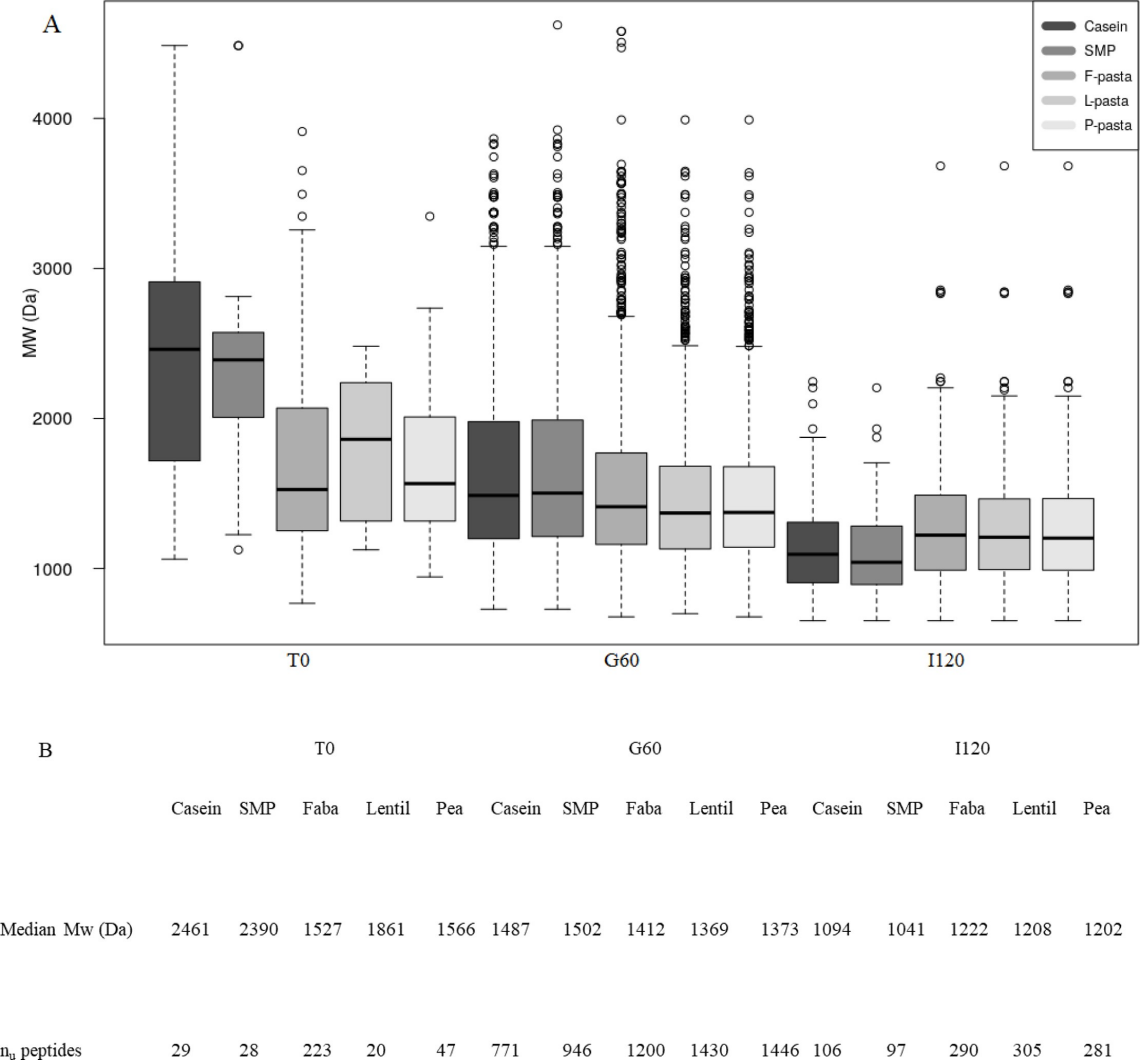
Boxplot of the molecular weights of unique peptides identified after 60 minutes of gastric digestion (G60) or 120 minutes of intestinal digestion (I120) compared to the initial digestion time (T0) for each type of diet digestate (Fig 4A). Table summarizing median molecular weight, the number of unique peptide sequences (n_u_) in each phase of digestion and for each type of diet digestate (Fig 4B). F-pasta or Faba = faba bean enriched pasta diet; L-pasta or Lentil = lentil enriched pasta diet; P-pasta or Pea = split pea enriched pasta diet; SMP = soluble milk protein diet; Casein = casein diet.

Peptidome analysis were undertaken to explore the impact of both the nature of the legume used to enrich the pasta and of the structure of the legume enriched pasta itself on the peptide profiles generated during digestion. The amino acid sequences of the soluble peptides present in each digestate at T0, G60 and I120 were determined. The method used in this study resulted in a window of identification of peptides between a maximum molecular weight of 4 kDa, to obtain efficient fragmentation in the collision cell and a minimum peptide length of 6 amino acids (AA), to obtain a sufficiently long peptide sequence for it to be unambiguously matched to a single protein sequence. Even if this ‘identification window’ was quite narrow, some trends emerged when we compared the number of unique identified peptides (n_u_) common to the two replicates of each digestion time and the median molecular weight of the peptide population (‘MW’) between samples (Fig 4A and 4B). Even though SDS Page analysis was conducted in reducing conditions, it resulted in a complete sample description by displaying 2.5 kDa to 200 kDa peptides and proteins.

Focusing on the comparison between milk enriched protein and legume enriched pasta diets at the initial time of digestion, we observed that milk enriched protein diets started with a higher molecular weight population than the pasta diets, with median MW of around 2.4 kDa versus 1.5–1.8 kDa, respectively. The number of unique peptides (n_u_) was low in all samples (n_u_ = 20–47). Only the F-pasta diet stood out with n_u_ = 223. Then, at the end of the gastric phase, the MW of milk protein and legume enriched pasta diets tightened. Compared with the beginning, MW distribution at the end of intestinal phase was reversed, with a lower MW in milk protein diets than in legume enriched pasta diets. At the end of intestinal phase, the three pasta diets also had more unique peptides (n_u_ = 281–305) than the milk protein diets (n_u_ = 97–106). These results led us to hypothesize that animal protein diets are more quickly and extensively digested than plant protein diets. SDS Page of milk protein diets confirmed our hypothesis, with no residual bands of peptide remaining at the end of intestinal phase from 2.5 to 4 kDa.

The main difference between the MW and n_u_ in the three legume enriched pasta diets was observed at T0 when the F-pasta diet contained eight to ten times more unique peptides (n_u_ = 223) and also a much wider MW range (from 650 to 3912 Da). At the end of the intestinal phase, the median MW, the range of MW and the number of unique peptides in all the pasta diets were quite similar.

**Fig 5:**
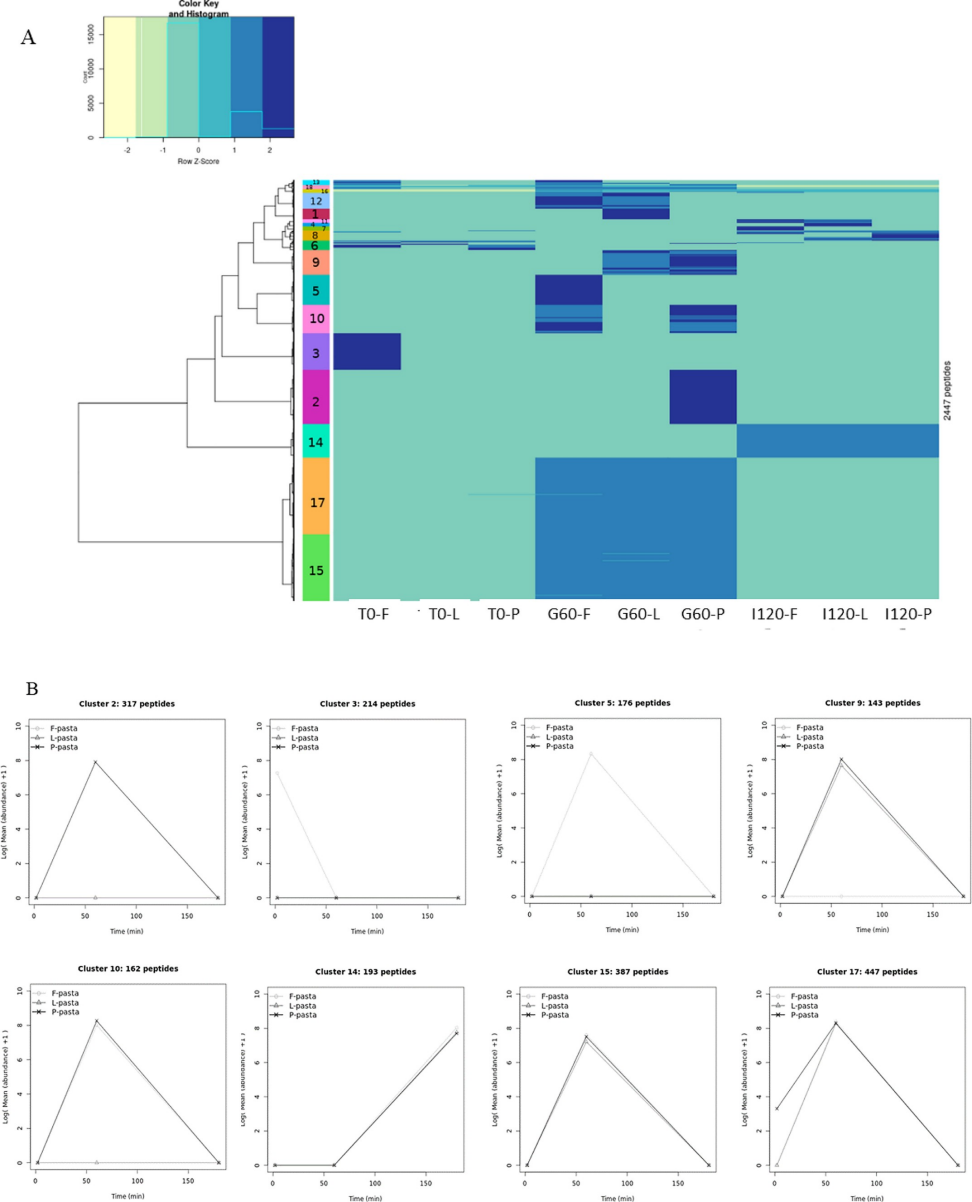
Clustering (Fig 5A) and number of unique peptides in each type of digestate in a given cluster (Fig 5B). T0-F = undigested faba bean enriched pasta diet; T0-L = undigested lentil enriched pasta diet; T0-P = undigested split pea enriched pasta diet; G60-F = digested faba bean enriched pasta diet after 60 minutes of gastric digestion; G60-L = digested lentil enriched pasta diet after 60 minutes of gastric digestion; G60-P = digested split pea enriched pasta diet after 60 minutes of gastric digestion; I120-F = digested faba bean enriched pasta diet after 120 minutes of intestinal digestion; I120-L = digested lentil enriched pasta diet after 120 minutes of intestinal digestion; I120-P = digested split pea enriched pasta diet after 120 minutes of intestinal digestion; F-pasta = faba bean enriched pasta diet; L-pasta = lentil enriched pasta diet; P-pasta = split pea enriched pasta diet.

**Fig 6:**
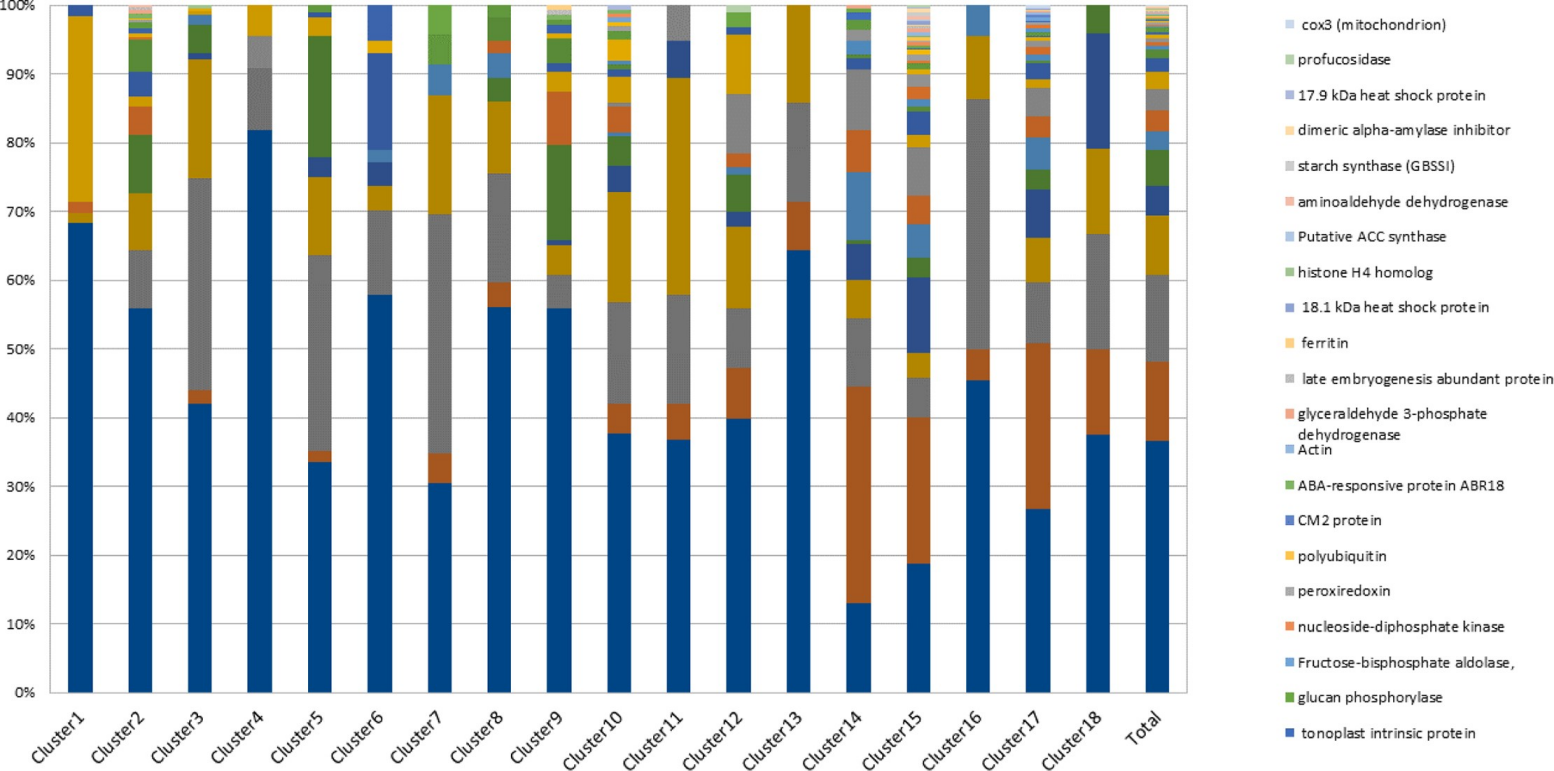
Contribution of parent proteins to the 18 clusters identified.

Cluster analysis grouped the 2,447 unique pasta diet peptide sequences in 18 clusters of specific behavior (Fig 5A). The parent proteins from which the released peptides derived were identified based on the published sequences of the proteins present in wheat and legume (split pea, faba bean, lentil) (S1 Table). However, as lentil and faba bean proteins were 10 times less numerous than split pea proteins in data banks, we cannot exclude a possible bias in the number of peptides identified. The proteins present in legumes and wheat were notably vicilin, HMW and LMW wheat glutenin proteins, alpha and gamma gliadins, legumin, albumin, and other proteins including antinutritional factors, i.e. lectin, enzymes or enzymatic inhibitor of enzymes (Fig 6). This overview of the origin of peptide proteins emphasized the fact that vicilin (37%), legumin B (12.6%), HMW glutenins (11.5%) and legumin A2 (8.5%) were the main contributors.

The relationship between the clusters and the proteins from which the quantified peptides originated was analyzed using a chi-square test (see Tables 4 and 5 for selected clusters and S2 and S3 Tables for complementary clusters). To know which protein was correlated with which cluster, another chi-square test was performed between categories. The intensity of the correlation of a given protein with another given cluster depended: 1) on the percentage of peptides in the given cluster that originated from the given protein (Cla/mod), 2) on the percentage of all the peptides originating from the given protein that belonged to the given cluster (mod.cla), and 3) on the percentage of all peptides arising from the given protein across all clusters (global).

**Table 4:** Relationship between the selected clusters and the parent protein of the unique peptides analyzed with the chi-square test (p < 0.05).

Cluster	Modality	Description	Cla/Mod^a^	Mod/Cla^b^	Global^c^	v.test	p.value
2	prot = a1	Vicilin	19.71	55.84	36.70	7.43	0.0000
	prot = b24	Albumin (pea)	51.72	4.73	1.19	5.00	0.0000
	prot = a9	p54	21.26	8.52	5.19	2.68	0.0074
	prot = a4	Legumin B	8.77	8.52	12.59	-2.42	0.0155
	prot = b12	Gamma Gliadin (wheat)	0.00	0.00	2.82	-4.01	0.0001
	prot = b15	HMW Glutenin (wheat)	0.00	0.00	2.98	-4.15	0.0000
	prot = a8	Alpha-Gliadin (wheat)	0.00	0.00	4.37	-5.16	0.0000
	prot = a3	LMW Glutenin (wheat)	0.00	0.00	11.52	-8.87	0.0000
3	prot = a4	Legumin B	21.43	30.84	12.59	7.44	0.0000
	prot = a7	Legumin A2	17.79	17.29	8.50	4.35	0.0000
	prot = b16	Lectin	1.67	0.47	2.45	-2.17	0.0304
	prot = b17	Dehydrin (pea)	0.00	0.00	2.13	-2.65	0.0081
	prot = a8	Alpha-Gliadin (wheat)	1.87	0.93	4.37	-2.91	0.0036
	prot = b15	HMW Glutenin (wheat)	0.00	0.00	2.98	-3.26	0.0011
	prot = b14	Lipoxygenase (pea)	0.00	0.00	3.11	-3.34	0.0009
	prot = a3	LMW glutenin (wheat)	1.42	1.87	11.52	-5.44	0.0000
5	prot = a9	p54	24.41	17.61	5.19	6.28	0.0000
	prot = a4	Legumin B	16.23	28.41	12.59	5.85	0.0000
	prot = b12	Gamma gliadin (wheat)	0.00	0.00	2.82	-2.78	0.0054
	prot = b15	HMW Glutenin (wheat)	0.00	0.00	2.98	-2.88	0.0040
	prot = b14	Lipoxygenase (pea)	0.00	0.00	3.11	-2.95	0.0031
	prot = a3	LMW Glutenin (wheat)	1.06	1.70	11.52	-5.00	0.0000
9	prot = a1	Vicilin	8.91	55.94	36.70	4.80	0.0000
	prot = a9	p54	15.75	13.99	5.19	4.14	0.0000
	prot = b14	Lipoxygenase (pea)	14.47	7.69	3.11	2.80	0.0051
	prot = b24	Albumin (pea)	17.24	3.50	1.19	2.17	0.0297
	prot = a7	Legumin A2	2.88	4.20	8.50	-2.00	0.0450
	prot = b12	Gamma Gliadin (wheat)	0.00	0.00	2.82	-2.44	0.0148
	prot = a8	Alpha-Gliadin (wheat)	0.93	0.70	4.37	-2.50	0.0123
	prot = b15	HMW Glutenin (wheat)	0.00	0.00	2.98	-2.53	0.0115
	prot = a4	Legumin B	2.27	4.90	12.59	-3.12	0.0018
	prot = a3	LMW Glutenin (wheat)	0.00	0.00	11.52	-5.67	0.0000
10	prot = a7	Legumin A2	12.50	16.05	8.50	3.24	0.0012
	prot = b29	Seed biotinylated protein of 65 kDa (pea)	33.33	3.09	0.61	3.05	0.0023
	prot = b15	HMW Glutenin (wheat)	1.37	0.62	2.98	-2.00	0.0458
	prot = a3	LMW Glutenin (wheat)	2.48	4.32	11.52	-3.26	0.0011
14	prot = a3	LMW Glutenin (wheat)	21.63	31.61	11.52	7.86	0.0000
	prot = b12	Gamma Gliadin (wheat)	27.54	9.84	2.82	4.95	0.0000
	prot = b15	HMW Glutenin (wheat)	23.29	8.81	2.98	4.12	0.0000
	prot = b25	Alpha-Amylase inhibitor. tetrameric. chain CM13 (wheat)	30.77	2.07	0.53	2.38	0.0174
	prot = b14	Lipoxygenase (pea)	15.79	6.22	3.11	2.33	0.0196
	prot = b38	Tonoplastic intrinsic protein (faba)	50.00	1.04	0.16	2.11	0.0352
	prot = b16	Lectin	0.00	0.00	2.45	-2.71	0.0068
	prot = a9	p54	0.79	0.52	5.19	-3.62	0.0003
	prot = a1	Vicilin	2.78	12.95	36.70	-7.64	0.0000
15	prot = a8	Alpha-Gliadin (wheat)	40.19	11.11	4.37	6.20	0.0000
	prot = a3	LMW Glutenin (wheat)	29.08	21.19	11.52	6.02	0.0000
	prot = b15	HMW Glutenin (wheat)	36.99	6.98	2.98	4.46	0.0000
	prot = b26	elongation factor	53.85	1.81	0.53	3.10	0.0019
	prot = b28	heat shock protein 70 (pea)	50.00	1.81	0.57	2.93	0.0034
	prot = b12	Gamma Gliadin (wheat)	27.54	4.91	2.82	2.51	0.0120
	prot = b56	Dimeric Alpha-Amylase Inhibitor (wheat)	100.00	0.52	0.08	2.24	0.0250
	prot = b55	Starch Synthase (wheat)	100.00	0.52	0.08	2.24	0.0250
	prot = b43	polyubiquin	100.00	0.52	0.08	2.24	0.0250
	prot = b37	Cu/Zn Superoxide Dismutase (pea)	60.00	0.78	0.20	2.13	0.0333
	prot = a9	p54	8.66	2.84	5.19	-2.38	0.0171
	prot = a7	Legumin A2	6.73	3.62	8.50	-4.07	0.0000
	prot = a4	Legumin B	7.14	5.68	12.59	-4.81	0.0000
	prot = a1	Vicilin	8.13	18.86	36.70	-8.26	0.0000
17	prot = a3	LMW Glutenin (wheat)	38.30	24.16	11.52	8.49	0.0000
	prot = a8	Alpha-Gliadin (wheat)	28.97	6.94	4.37	2.77	0.0057
	prot = b34	Alpha-Amylase inhibitor. tetrameric. chain CM16 (wheat)	100.00	0.67	0.12	2.74	0.0061
	prot = b12	Gamma Gliadin (wheat)	30.43	4.70	2.82	2.49	0.0129
	prot = b59	COX 3 mitochondrion (wheat)	100.00	0.45	0.08	2.13	0.0333
	prot = b44	CM2 (protein (wheat)	100.00	0.45	0.08	2.13	0.0333
	prot = a9	p54	10.24	2.91	5.19	-2.52	0.0117
	prot = a4	Legumin B	12.99	8.95	12.59	-2.64	0.0083
	prot = a1	Vicilin	13.25	26.62	36.70	-4.97	0.0000

A parent protein of a unique vegetable source is referred to by name of the protein

^a^Cla/Mod is the occurrence of the modality category in the cluster divided by its occurrence in the entire dataset

^b^Mod/Cla is the proportion of the modality category within the cluster

^c^Global is the global proportion of this modality category within the entire dataset.

**Table 5:** Relationship between the selected clusters and some quantitative modalities (some amino-acid contents and digestion times) analyzed by a chi-square test (p < 0.05).

Cluster	Modality	Mean ± SD per category			Global mean ± SD			v.test	p.value
2	G60-P	7.49	±	0.62	4.60	±	3.68	14.98	0.0000
	T0-L	0.00	±	0.00	0.07	±	0.66	-2.06	0.0390
	T0-P	0.00	±	0.00	0.15	±	0.96	-2.98	0.0029
	I120-P	0.00	±	0.00	0.78	±	2.23	-6.63	0.0000
	I120-F	0.00	±	0.00	0.83	±	2.36	-6.74	0.0000
	I120-L	0.00	±	0.00	0.82	±	2.31	-6.81	0.0000
	Glutamine	0.95	±	1.18	2.13	±	3.20	-7.04	0.0000
	T0-F	0.00	±	0.00	0.85	±	2.28	-7.14	0.0000
	G60-L	0.00	±	0.00	3.54	±	3.71	-18.19	0.0000
	G60-F	0.00	±	0.00	4.15	±	3.82	-20.72	0.0000
3	T0-F	6.88	±	0.57	0.85	±	2.28	40.5	0.0000
	Leucine	1.33	±	0.95	0.90	±	0.86	7.71	0.0000
	T0-P	0.00	±	0.00	0.15	±	0.96	-2.39	0.0170
	Glutamine	1.51	±	2.11	2.13	±	3.20	-2.96	0.0031
	I120-P	0.00	±	0.00	0.78	±	2.23	-5.32	0.0000
	I120-F	0.00	±	0.00	0.83	±	2.36	-5.41	0.0000
	I120-L	0.00	±	0.00	0.82	±	2.31	-5.46	0.0000
	G60-L	0.00	±	0.00	3.54	±	3.71	-14.59	0.0000
	G60-F	0.00	±	0.00	4.15	±	3.82	-16.63	0.0000
	G60-P	0.00	±	0.00	4.60	±	3.68	-19.16	0.0000
5	G60-F	7.79	±	0.71	4.15	±	3.82	13.11	0.0000
	T0-P	0.00	±	0.00	0.15	±	0.96	-2.15	0.0320
	Glutamine	1.57	±	2.51	2.13	±	3.20	-2.4	0.0160
	I120-P	0.00	±	0.00	0.78	±	2.23	-4.78	0.0000
	I120-F	0.00	±	0.00	0.83	±	2.36	-4.86	0.0000
	I120-L	0.00	±	0.00	0.82	±	2.31	-4.91	0.0000
	T0-F	0.00	±	0.00	0.85	±	2.28	-5.15	0.0000
	G60-L	0.00	±	0.00	3.54	±	3.71	-13.12	0.0000
	G60-P	0.00	±	0.00	4.60	±	3.68	-17.23	0.0000
9	G60-L	7.09	±	0.78	3.54	±	3.71	11.79	0.0000
	G60-P	7.51	±	0.70	4.60	±	3.68	9.75	0.0000
	I120-P	0.00	±	0.00	0.78	±	2.23	-4.28	0.0000
	I120-F	0.00	±	0.00	0.83	±	2.36	-4.35	0.0000
	I120-L	0.00	±	0.00	0.82	±	2.31	-4.4	0.0000
	T0-F	0.00	±	0.00	0.85	±	2.28	-4.61	0.0000
	Glutamine	0.79	±	1.15	2.13	±	3.20	-5.17	0.0000
	G60-F	0.00	±	0.00	4.15	±	3.82	-13.38	0.0000
10	G60-F	7.19	±	0.89	4.15	±	3.82	10.5	0.0000
	G60-P	7.44	±	0.88	4.60	±	3.68	10.17	0.0000
	T0-P	0.00	±	0.00	0.15	±	0.96	-2.06	0.0400
	I120-P	0.00	±	0.00	0.78	±	2.23	-4.57	0.0000
	I120-F	0.00	±	0.00	0.83	±	2.36	-4.65	0.0000
	I120-L	0.00	±	0.00	0.82	±	2.31	-4.7	0.0000
	T0-F	0.00	±	0.00	0.85	±	2.28	-4.93	0.0000
	G60-L	0.00	±	0.00	3.54	±	3.71	-12.55	0.0000
14	I120-F	7.53	±	0.68	0.83	±	2.36	41.06	0.0000
	I120-P	7.09	±	0.80	0.78	±	2.23	40.93	0.0000
	I120-L	7.26	±	0.68	0.82	±	2.31	40.25	0.0000
	Glutamine	3.17	±	3.16	2.13	±	3.20	4.69	0.0000
	T0-P	0.00	±	0.00	0.15	±	0.96	-2.26	0.0240
	Leucine	0.71	±	0.73	0.90	±	0.86	-3.24	0.0012
	T0-F	0.00	±	0.00	0.85	±	2.28	-5.42	0.0000
	G60-L	0.00	±	0.00	3.54	±	3.71	-13.8	0.0000
	G60-F	0.00	±	0.00	4.15	±	3.82	-15.72	0.0000
	G60-P	0.00	±	0.00	4.60	±	3.68	-18.11	0.0000
15	G60-L	6.79	±	0.60	3.54	±	3.71	18.77	0.0000
	G60-F	7.14	±	0.59	4.15	±	3.82	16.79	0.0000
	G60-P	6.91	±	0.66	4.60	±	3.68	13.45	0.0000
	Glutamine	3.57	±	4.21	2.13	±	3.20	9.62	0.0000
	T0-L	0.00	±	0.00	0.07	±	0.66	-2.32	0.0200
	T0-P	0.00	±	0.00	0.15	±	0.96	-3.35	0.0008
	I120-P	0.00	±	0.00	0.78	±	2.23	-7.45	0.0000
	I120-F	0.00	±	0.00	0.83	±	2.36	-7.57	0.0000
	I120-L	0.00	±	0.00	0.82	±	2.31	-7.65	0.0000
	T0-F	0.00	±	0.00	0.85	±	2.28	-8.02	0.0000
17	G60-L	7.89	±	0.54	3.54	±	3.71	27.41	0.0000
	G60-F	8.02	±	0.55	4.15	±	3.82	23.7	0.0000
	G60-P	7.89	±	0.53	4.60	±	3.68	20.92	0.0000
	Glutamine	2.78	±	3.74	2.13	±	3.20	4.77	0.0000
	T0-L	0.00	±	0.00	0.07	±	0.66	-2.53	0.0110
	T0-P	0.03	±	0.38	0.15	±	0.96	-3.04	0.0024
	I120-P	0.00	±	0.00	0.78	±	2.23	-8.12	0.0000
	I120-F	0.00	±	0.00	0.83	±	2.36	-8.26	0.0000
	I120-L	0.00	±	0.00	0.82	±	2.31	-8.34	0.0000
	T0-F	0.00	±	0.00	0.85	±	2.28	-8.75	0.0000

Based on this statistical analysis, we decided to focus on eight out of the 18 clusters, representing 83% of the total number of detected peptides. These eight clusters (clusters: 2, 3, 5, 9, 10, 14, 15 and 17) were all strongly correlated with digestion times (Fig 5B, Table 5).

T0 of F-pasta diet was highly correlated with cluster 3 (Tables 4 and 5) which represented 9% of the total unique peptides (n_u_ = 214 out of n_u_ = 2,447). This cluster was highly correlated with legume storage proteins: legumin B and to a lesser extent with legumin A2, originated for 85% and 94% from faba bean, respectively (sub-group analysis not shown). Cluster 3 was not correlated to G60 nor to I120 of any pasta diets (Fig 5B). In addition, peptides of cluster 3 were particularly rich in leucine (Tables 4 and 5).

Gastric times were correlated with big clusters (2, 5, 9, 10, 15 and 17) (Fig 5A and 5B, Tables 4 and 5). These clusters grouped the largest number of unique peptides (75% of total), indicating that the gastric phase is an important phase in differentiating the pattern of peptides. If we look for similarities between pasta diets, all the G60 are correlated with clusters 15 and 17 (representing n_u_ = 834, i.e. 34% of the total unique peptides n_u_ = 2,447) and negatively correlated with T0 and intestinal samples. In clusters 15 and 17 (834 unique peptides), we mainly found peptides derived from wheat proteins, such as LMW glutenin, gamma and alpha gliadins representing 67%, 58% and 69% of the unique peptides originating from this protein (n_u_ = 282, 69 and 107, respectively). HMW glutenins were only highly correlated with cluster 15: 37% of the unique peptides originating from this protein (n_u_ = 73) were in this cluster. Legume storage proteins such as vicilin and to a lesser extent legumins (A2, B) were negatively correlated with clusters 15 and 17. These clusters were qualitatively correlated with peptides rich in glutamine. Conversely, if we look at the difference between the three pasta diets, G60 in the P-pasta diet was significantly correlated with cluster 2 (representing 13% of the total unique peptides, i.e. n_u_ = 317), in which 56% of the peptides originated from vicilin among which 82% are from pea (results not shown). G60 in the F-pasta diet was highly correlated with cluster 5 (representing 7% of the total unique peptides, i.e. n_u_ = 176) which was distinguished by the presence of a high proportion of peptides originating from P54 (18% of the unique peptides of the cluster n_u_ = 176). Fewer amounts of such peptides were found in G60 originating from L- and P-pasta diets. These diets were correlated with cluster 9 (6% of the total unique peptide, i.e. n_u_ = 143) positively correlated itself with vicilin, P54, lipoxygenase and albumin. Cluster 9 was negatively correlated with G60 originating from the F-pasta diet again underlining a difference in the peptide composition of G60 of F-pasta diet compared to the G60 of the two other legume enriched pasta diets. However, G60 originating from F-pasta with P-pasta diets were both correlated with cluster 10, both accounting for 7% of the total unique peptides, i.e. n_u_ = 162. This cluster was positively correlated with a seed biotinylated protein and to a lesser extent with legumin A2.

The intestinal phase of the three pasta diets was highly correlated with cluster 14, itself correlated with peptides derived from wheat (Fig 5A and 5B, Tables 4 and 5). This cluster grouped 193 peptides, i.e. 8% of the total unique peptides. The cluster typically contained peptides rich in glutamine originating from several wheat proteins including gamma gliadin, LMW and HMW glutenins. More precisely, 32% of the 193 peptides of this cluster originated from LMW glutenin. Cluster 14 was also negatively correlated with gastric times. This indicates that new specific peptides are released from wheat at these intestinal times. The fact that this cluster is correlated with all three types of pasta diets reveals that the release of peptides from wheat in the intestinal phase occurs independently of the type of legume enrichment in the pasta diets and mainly originate from LMW glutenins.

Altogether the 2,447 peptides generated during the digestion of legume enriched pasta could be grouped into 18 clusters among which 8 clusters summarized 83% of the peptides. The cluster 3 underlined the specificity of peptide composition of faba bean-enriched pasta at T0. Six clusters gathering 75% of the peptides were correlated with gastric phase indicating the important differentiation of peptides pattern for the three pasta during this digestion phase. Indeed, if all pasta developed during this phase LMW glutenin, alpha and gamma-gliadin peptides some specificities emerged: P-pasta released peptides from vicilin whereas F-pasta released P54, lipoxygenase and albumin peptides. Considering intestinal time, the cluster 14 was correlated both with specific peptides originated from wheat proteins (gamma gliadin, LMW and HMW glutenins) and was anti-correlated to gastric time for all pasta, it suggests that the nature of legume enrichment does not modify the action of intestinal proteases.

## Discussion

The primary objective of this study was to evaluate the effect of varying legume sources (split pea, lentil or faba bean) on protein digestion kinetics of pasta diets made from legume and wheat compared with milk protein diets.

### Pasta diet qualities and digestion kinetics compared to milk protein diets

All pasta were enriched with legume flour in order to reach 21% of protein content, which is 1.6 fold higher than that of 100% wheat pasta [24]. Thus, 100 g of cooked legume enriched pasta represented about 13% of the recommended daily protein intake for a healthy young subject weighing 70 kg. Thanks to the complementary essential amino acid composition of legume and wheat [6], the essential amino acid contents of F-, L- and P-pasta diets were higher than those recommended for human adults by WHO/FAO/UNU [26] indicating that such enrichment is a good way to achieve a diet with balanced amino acids. Globally, the essential amino acid composition of the three legume enriched pasta diets was close. Compared with pasta only made with wheat (13% protein content), our legume enriched pasta diets were richer in all essential amino acids except sulfur amino acids (about 8% lower) well represented in cereals [24]. However, their essential amino acid contents were lower than the ones in the casein and SMP diets which were notably richer in branched chain amino acids i.e. leucine, valine and isoleucine and sulfur amino acids of interest for tissue growth and for the prevention of catabolic actions during exercise [53]. Lysine was also much more concentrated in milk protein diets. In addition to the amino acid balance, the enrichment of pasta with legume flour also increased its fiber content especially the L- and P-pasta diets (10.4 and 11.7%, respectively versus 3.1 for a 100% wheat pasta, [8]). The specific richness in fibers of lentil pasta in comparison to faba bean pasta was underlined by Laleg et al. [21] in 100% lentil pasta and was due to residual cell wall structures rich in cellulose and hemicellulose. Of course, these fibers are important prebiotics that help balance gastro-intestinal microflora and have a vast array of health benefits [54].

Concerning the kinetics of digestion of legume enriched pasta diets and milk protein diets and according to the degree of hydrolysis and peptidome analysis, casein and SMP diets were hydrolyzed faster and to a greater extent than legume enriched pasta diets during the gastric and intestinal phases. Their degrees of proteolysis were 1.2 and 1.4-fold higher than for legume enriched pasta diets at the end of the gastric and intestinal phases, respectively. According to peptidome analysis, there were 43% and 66% fewer unique peptides in milk protein diets than in legume enriched pasta protein diets at the end of gastric and intestinal phases, respectively, also suggesting that milk protein diets hydrolyze more rapidly and more completely than legume enriched pasta diets. These results are in accordance with those of Nguyen et al. [55] who demonstrated higher levels of hydrolysis for dairy proteins than for soy proteins during in vitro digestion. Similarly, Savoie et al. [56] compared the kinetics of peptide release during in vitro digestion of purified casein, soy protein isolate and wheat gluten, and observed a higher rate of digestion of animal proteins. The difference in the degree of proteolysis between animal proteins and plant proteins can be explained by several factors corresponding to the different levels of protein structure. First, protein interactions within the protein network could at least partially affect protein digestibility. Regarding our SE-HPLC analysis, the protein fraction of legume enriched pasta diets that is soluble in SDS+DTE was 28-fold higher than that of casein and SMP diets. This means that legume enriched pasta protein diets were more covalently linked (by S-S bonds) than casein and SMP diets, which could reduce their susceptibility to proteolysis. Note that the difference in protein digestibility between animal diets and legume enriched pasta diets was clearer during intestinal digestion with pancreatic enzymes (trypsin, chymotrypsin) than during gastric digestion with pepsin. This is likely at least partially due to the presence of residual trypsin inhibitors, even though these are greatly reduced by processing [6,57]. At the level of primary protein structure, the number of cleavage sites specific to enzyme digestion is also a key determinant of protein digestibility [58]. The preferential cleavage of peptide bonds by pepsin are leucine, phenylalanine, tryptophan and tyrosine residues [59]. Trypsin cleaves peptide bonds at the carboxyl side of arginine or lysine, except when these residues are linked to a proline residue, which causes steric hindrance and hinders the action of the enzyme [60]. Chymotrypsin catalyzes the hydrolysis of peptide bonds on the carboxyl side of the phenylalanine, tryptophan and tyrosine residues and also hydrolyzes peptide bonds formed by methionine, leucine and histidine residues at a slower rate [59]. Lysine, methionine, leucine and tryptophan were from 1.1 to 3.5 times more concentrated in casein and SMP diets than in legume enriched pasta diets. Thus, milk protein diets have more cleavage sites specific to pepsin, trypsin and chymotrypsin contained in pancreatic enzyme at least partially explaining their higher degree of proteolysis.

### Digestion kinetics of the legume enriched pasta diets as a function of the legume used for enrichment

At the end of the gastric phase, the degree of hydrolysis (about 6%) of legume enriched pasta diets were comparable regardless of the type of legume, as was their electrophoretic peptide patterns gathering peptides from 2.5 to 10 kDa. The low percentage of protein hydrolysis reached after the gastric phase in all the legume enriched pasta is in agreement with the results of a previous study [24]. In pasta enriched with 35% legume, Laleg et al. [24] also reported that only 6.4% of total proteins were degraded by pepsin in the gastric phase after 30 min of digestion. According to the literature, the viscosity of legume proteins increases significantly at gastric pH, as it is close to the isoelectric pH of legume protein (pH = 4) [61–63] which could hinder the diffusion of pepsin in the digestate and its subsequent action regardless of the type of protein interaction at molecular scale. Pepsin preferentially acts on peptide bonds located between hydrophobic and aromatic residues such as phenylalanine, tryptophan and tyrosine. As most of these residues are quite homogeneously distributed in legume enriched pasta diets, the difference between the number of theoretical cleavage sites of pepsin in the different pasta diets could be expected to be minimal. At the end of intestinal phase, differences in the degree of hydrolysis between pasta diets were observed, the F-pasta diet being more hydrolyzed than the L- and P-pasta diets based on the ninhydrin test. This difference was not detectable with SDS Page nor with peptidome analysis, as the proteolysis of F-pasta diet probably generated low molecular weight peptides and free amino acids.

Peptidome analysis completed the description of legume enriched pasta digestibility by detecting peptides of 6 AA to 4 kDa during the course of digestion. Not surprisingly, most of the unique peptides were derived from vicilin (also known as 7S globulin) giving rise to 37% of total peptides and also known to contain no disulfide bond and to be more susceptible to proteolytic processes than other globulins [64]. In addition to LMW wheat glutenin, other globulins B and A2 (also known as 11S globulins), were also important contributors (33% all together) of digestive peptidomes. The widely accepted model for 11S globulin is still that proposed for *Vicia faba* legumin [65]. In this model, the 11S globulin appears as a densely packed protein which could at least partially explain their low degree of hydrolysis. Peptide analysis also indicated that legume derived peptides differed in the three legume enriched pasta diets. Notably, peptide clustering analysis helped distinguish F-pasta protein diet from L- and P-pasta diets, indicating higher pre-proteolysis in F-pasta (also corroborated by the SDS Page profile) and the presence of a high proportion of peptides originating from P54 at the end of the gastric phase. The initial peptides in the F-pasta probably degraded faster than in L- and P-pasta since they do not accumulate and led to similar average MW and number of peptides at the end of the gastric times in all the legume enriched pasta digestates. The nature of the legume used to enrich pasta did not influence the pattern of peptides released from wheat proteins. Indeed, wheat derived peptide patterns were quite similar in all three legume enriched pasta diet digestates during the gastric and intestinal phases of protein digestion. This suggests that limited interactions occurred between legume and wheat proteins, inducing a similar proteolytic susceptibility of wheat proteins regardless of the legume, which probably act as fillers in the wheat pasta matrix network. Moreover, depending on the digestion time, new specific peptides were released from wheat proteins i.e. gamma gliadin, HMW and mainly LMW glutenins at the end of the intestinal phase, indicating that wheat proteins were mainly hydrolyzed at this phase of digestion. On the contrary, legume storage proteins were hydrolyzed more rapidly i.e. during the gastric phase and are likely less resistant to proteolysis than wheat proteins.

When considering difference in intestinal kinetics between legume enriched pasta diets, we hypothesize that the structure of the protein network is a key determinant. Indeed, the protein network of F-pasta diet that reached the highest extent of hydrolysis were less covalently bonded than the L- and P-pasta diets. Conversely, the amino acid composition of the different pasta diets was comparable and there is high amino-acids sequence conservation for storage proteins in legumes [64], and thus we expected minimal differences in the number of theoretical enzyme cleavage sites between the different pasta diets. Moreover, F-pasta diet contained the lowest fiber content and the lowest TIA content, both elements have been shown in plant derived matrices to reduce their protein digestibility by preventing enzymes from accessing proteins [66,67].

There is currently no available data on the peptidome of legume enriched pasta digestates despite the interest and development of foodomics. However, a few studies have assessed the in vitro protein digestibility of various legume enriched pasta by evaluating their degree of proteolysis in 5–30% lupin flour enriched pasta [13], 10–70% faba bean flour enriched pasta [22,24,25] and 20% pigeon pea flour enriched pasta [68]. These authors reported more extensive gastrointestinal digestion of legume enriched pasta (range 46% to 52%) compared to classical wheat pasta (42%, [22]), which is in agreement with our data. However, the detailed extent of hydrolysis reported in the literature may vary. For 70% faba bean enriched pasta, Laleg et al. [22] reported a slightly lower degree of hydrolysis (48%) at the end of the intestinal phase than the value obtained in our study for 62% faba bean enriched pasta diet (58%). This could be explained by the digestion protocol used by Laleg et al. [22] with lower gastric and intestinal enzyme concentrations and no preliminary digestion with salivary amylase. A preliminary degradation of starch, notably during the oral phase, has indeed been demonstrated to facilitate the access of peptidase for protein cleavage [69,70]. Rizzello et al. [25] reported higher proteolysis values for 50% faba bean enriched pasta (75.2%) than those obtained in our study. Again, a difference in digestion procedure could explain this variation.

## Conclusions

In conclusion, milk protein diets, which have a weaker protein network structure and more cleavage sites that are specific to digestive enzymes, were hydrolyzed faster and to a greater extent than legume enriched pasta. In addition, the different legumes used to enrich pasta had a significant impact on their protein network structure and therefore on legume protein degradation kinetics. Indeed, faba bean enriched pasta had the weakest protein network, the fastest digestion kinetics and the highest digestion rate with a discriminating peptide profile since the early times of digestion. The gastric phase of digestion allowed us to distinguish patterns of legume-derived peptides between pasta: vicilin, legumin B and P54 are the three most important contributors of digestive peptidomes in legume enriched pasta. The wheat proteins in legume-protein pasta were mainly digested in the intestinal phase and their digestive behavior was similar regardless of the type of legume (split pea, lentil or faba bean) used to enrich pasta as evidenced by peptidome analysis. This suggests that legume proteins did not strongly interfere with wheat protein in legume enriched pasta, acting therefore as a bulking agent in the gluten network.

It will be interesting to evaluate in vivo the differences in protein digestion observed in our study to assess the impact of legume enriched pasta in optimizing food intake in young healthy and older populations who require a high-quality food in comparison with milk proteins.

## Supporting information

S1 Fig Electrophoretic patterns (T = 12%) under reducing conditions of undigested wheat semolina, F-flour, L-flour and P-flour. F-flour = faba bean flour; L-flour = lentil flour; P-flour = split pea flour.
(PDF)

S2 Fig Protein aggregation of legume flour by SE-HPLC. Different letters represent significant difference between groups (p < 0.05). F-flour = faba bean flour; L-flour = lentil flour; P-flour = split pea flour.
(PDF)

S1 Table Contribution of parent protein of unique peptides within clusters and diet peptidome. (PDF)

S2 Table Relationship between the 18 clusters and the parent protein of the unique peptides analyzed with the chi-square test (p < 0.05). The parent protein of a unique vegetable source is referred to with name of the protein in parentheses with Cla/Mod^a^: occurrence of the modality category in the cluster divided by its occurrence in the entire dataset; Mod/Cla^b^: proportion of the modality category within the cluster; Global^c^: global proportion of this modality category within the entire dataset.
(PDF)

S3 Table Relationship between the 18 clusters and some quantitative modalities (some amino-acid contents and digestive times) analyzed with the chi-square test (p < 0.05). (PDF)

S1 Raw image (PDF)
