# Supplementary material for: Effect of protein aggregation in wheat-legume mixed pasta diets on their in vitro digestion kinetics in comparison to “rapid” and “slow” animal proteins
Source: PLoS One. 2020 May 4;15(5):e0232425. doi: 10.1371/journal.pone.0232425 (PMC7197814; doi:10.1371/journal.pone.0232425)
Supplement: S1 Fig — F-flour = faba bean flour; L-flour = lentil flour; P-flour = split pea flour. (PDF) [file pone.0232425.s001.pdf]

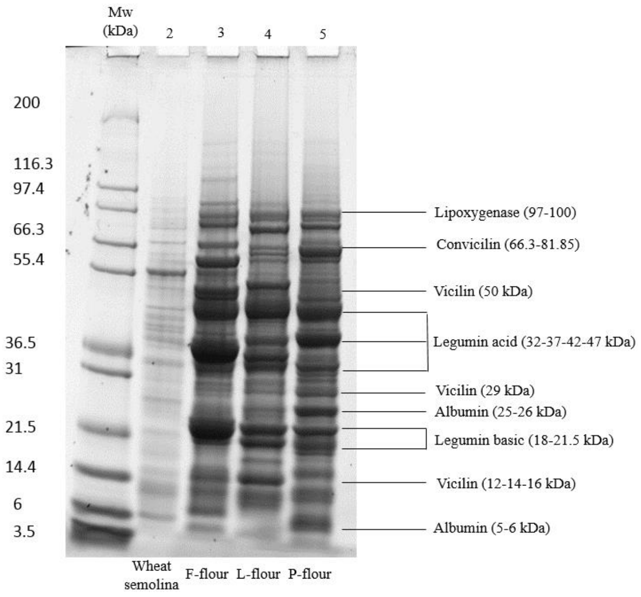

S1 Fig. Electrophoretic patterns (T = 12%) under reducing conditions of undigested wheat semolina, F-flour, L-flour and P-flour. F-flour = faba bean flour; L-flour = lentil flour; P-flour = split pea flour.
