# Supplementary material for: Effect of protein aggregation in wheat-legume mixed pasta diets on their in vitro digestion kinetics in comparison to “rapid” and “slow” animal proteins
Source: PLoS One. 2020 May 4;15(5):e0232425. doi: 10.1371/journal.pone.0232425 (PMC7197814; doi:10.1371/journal.pone.0232425)
Supplement: S1 Table — (PDF) [file pone.0232425.s003.pdf]

S1 Table. Contribution of parent protein of unique peptides within clusters and diet peptidome.

Description	protein code	Cluster																		Total
		1	2	3	4	5	6	7	8	9	10	11	12	13	14	15	16	17	18	
Vicilin	a1	43	177	90	18	59	33	7	32	80	61	7	37	18	25	73	10	119	9	**898**
LMW glutenin wheat	a3			4		3		1	2		7	1	7	2	61	82	1	108	3	**282**
Legumin B	a4		27	66	2	50	7	8	9	7	24	3	8	4	19	22	8	40	4	**308**
Legumin A2	a7	1	26	37		20	2	4	6	6	26	6	11	4	11	14	2	29	3	**208**
Alpha gliadin	a8			2		5	2			1	6	1	2		10	43		31	4	**107**
P54	a9		27	9		31			2	20	7		5		1	11		13	1	**127**
Gamma gliadin	b12			3			1	1	2		1		1		19	19	1	21		**69**
Lipoxygenase	b14	1	13						1	11	6		2		12	16		14		**76**
HMW Glutenin	b15				1						1	1	8		17	27		18		**73**
Lectin	b16	17	5	1	1	5				4	6		8			7		6		**60**
Dehydrin	b17	1	11			1	8			2	2		1		3	13		10		**52**
Albumin	b24		15						2	5	1				1	3		2		**29**
Alpha-amylase inhibitor, tetrameric, chain CM13	b25										1				4	4		4		**13**
Elongation factor	b26		1													7		5		**13**
Heat shock protein 70	b28														3	7		4		**14**
Seed biotinylated protein of 65 kDa	b29		2	1			1			1	5					3		2		**15**
Albumin 1	b30		2				3			2								1		**8**
Alcohol dehydrogenase	b31		3			2		1	1	1	2				3	3		2		**18**
Alpha-amylase inhibitor, tetrameric, chain CM16	b34																	3		**3**
Peptidyl-prolyl cis-trans isomerase	b35															2		2		**4**
Glycogen (starch) synthase,	b36		1								1					3		2		**7**
Cu/Zn superoxide dismutase	b37		1								1					3				**5**
Tonoplast intrinsic protein	b38														2	1		1		**4**
Glucan phosphorylase	b39							1					2		1	1				**5**
Fructose-bisphosphate aldolase,	b40										1							2		**3**
Nucleoside-diphosphate kinase	b41										1					2				**3**
Peroxiredoxin	b42															1				**1**
Polyubiquitin	b43															2				**2**
CM2 protein	b44																	2		**2**
ABA-responsive protein ABR18	b45		2							1	1					1				**5**
Actin	b46															2		1		**3**
Glyceraldehyde 3-phosphate Dehydrogenase	b47		2												1	2		1		**6**
Late embryogenesis abundant protein	b48		1							1						1				**3**
Ferritin	b49									1						1		1		**3**
18.1 kDa heat shock protein	b50															2		1		**3**
Histone H4 homolog	b51			1																**1**
Putative ACC synthase	b53															1				**1**
Aminoaldehyde dehydrogenase	b54		1													2				**3**
Starch synthase (GBSSI)	b55															2				**2**
Dimeric alpha-amylase inhibitor	b56															2				**2**
17.9 kDa heat shock protein	b57										1					1				**2**
Profucosidase	b58												1			1				**2**
Cox3 (mitochondrion)	b59																	2		**2**
**Total**		**63**	**317**	**214**	**22**	**176**	**57**	**23**	**57**	**143**	**162**	**19**	**93**	**28**	**193**	**387**	**22**	**447**	**24**	**2447**
