# Supplementary material for: Effect of protein aggregation in wheat-legume mixed pasta diets on their in vitro digestion kinetics in comparison to “rapid” and “slow” animal proteins
Source: PLoS One. 2020 May 4;15(5):e0232425. doi: 10.1371/journal.pone.0232425 (PMC7197814; doi:10.1371/journal.pone.0232425)
Supplement: S2 Table — The parent protein of a unique vegetable source is referred to with name of the protein in parentheses with Cla/Moda: occurrence of the modality category in the cluster divided by its occurrence in the entire dataset; Mod/Clab: proportion of the modality category within the cluster; Globalc: global proportion of this modality category within the entire dataset. (PDF) [file pone.0232425.s004.pdf]

**S2 Table A. Relationship between the 18 clusters and the parent protein of the unique peptides analyzed with the chi-square test ($p < 0.05$).**

| Cluster | Modality | Description | Cla/Mod[a] | Mod/Cla[b] | Global[c] | v.test | p.value |
|---|---|---|---|---|---|---|---|
| 1 | prot=b16 | Lectin | 28.33 | 26.98 | 2.45 | 7.67 | 0.0000 |
| | prot=a1 | Vicilin | 4.79 | 68.25 | 36.70 | 5.11 | 0.0000 |
| | prot=a9 | p54 | 0.00 | 0.00 | 5.19 | -2.13 | 0.0333 |
| | prot=a7 | Legumin A2 | 0.48 | 1.59 | 8.50 | -2.20 | 0.0276 |
| | prot=a3 | LMW glutenin (wheat) | 0.00 | 0.00 | 11.52 | -3.54 | 0.0004 |
| | prot=a4 | Legumin B | 0.00 | 0.00 | 12.59 | -3.74 | 0.0002 |
| 2 | prot=a1 | Vicilin | 19.71 | 55.84 | 36.70 | 7.43 | 0.0000 |
| | prot=b24 | Albumin (pea) | 51.72 | 4.73 | 1.19 | 5.00 | 0.0000 |
| | prot=a9 | p54 | 21.26 | 8.52 | 5.19 | 2.68 | 0.0074 |
| | prot=a4 | Legumin B | 8.77 | 8.52 | 12.59 | -2.42 | 0.0155 |
| | prot=b12 | Gamma Gliadin (wheat) | 0.00 | 0.00 | 2.82 | -4.01 | 0.0001 |
| | prot=b15 | HMW Glutenin (wheat) | 0.00 | 0.00 | 2.98 | -4.15 | 0.0000 |
| | prot=a8 | alpha-Gliadin (wheat) | 0.00 | 0.00 | 4.37 | -5.16 | 0.0000 |
| | prot=a3 | LMW Glutenin (wheat) | 0.00 | 0.00 | 11.52 | -8.87 | 0.0000 |
| 3 | prot=a4 | Legumin B | 21.43 | 30.84 | 12.59 | 7.44 | 0.0000 |
| | prot=a7 | Legumin A2 | 17.79 | 17.29 | 8.50 | 4.35 | 0.0000 |
| | prot=b16 | Lectin | 1.67 | 0.47 | 2.45 | -2.17 | 0.0304 |
| | prot=b17 | Dehydrin (pea) | 0.00 | 0.00 | 2.13 | -2.65 | 0.0081 |
| | prot=a8 | Alpha-Gliadin (wheat) | 1.87 | 0.93 | 4.37 | -2.91 | 0.0036 |
| | prot=b15 | HMW Glutenin (wheat) | 0.00 | 0.00 | 2.98 | -3.26 | 0.0011 |
| | prot=b14 | Lipoxygenase (pea) | 0.00 | 0.00 | 3.11 | -3.34 | 0.0009 |
| | prot=a3 | LMW Glutenin (wheat) | 1.42 | 1.87 | 11.52 | -5.44 | 0.0000 |
| 4 | prot=a1 | Vicilin | 2.00 | 81.82 | 36.70 | 4.27 | 0.0000 |
| 5 | prot=a9 | p54 | 24.41 | 17.61 | 5.19 | 6.28 | 0.0000 |
| | prot=a4 | Legumin B | 16.23 | 28.41 | 12.59 | 5.85 | 0.0000 |
| | prot=b12 | Gamma Gliadin (wheat) | 0.00 | 0.00 | 2.82 | -2.78 | 0.0054 |
| | prot=b15 | HMW Glutenin (wheat) | 0.00 | 0.00 | 2.98 | -2.88 | 0.0040 |
| | prot=b14 | Lipoxygenase (pea) | 0.00 | 0.00 | 3.11 | -2.95 | 0.0031 |
| | prot=a3 | LMW Glutenin (wheat) | 1.06 | 1.70 | 11.52 | -5,00 | 0.0000 |
| 6 | prot=b17 | Dehydrin (pea) | 15.38 | 14.04 | 2.13 | 4.27 | 0.0000 |
| | prot=b30 | Albumin 1 (pea, lentil) | 37.50 | 5.26 | 0.33 | 3.42 | 0.0006 |
| | prot=a1 | Vicilin | 3.67 | 57.89 | 36.70 | 3.26 | 0.0011 |
| | prot=a9 | p54 | 0.00 | 0.00 | 5.19 | -1.99 | 0.0462 |
| | prot=a3 | LMW Glutenin (wheat) | 0.00 | 0.00 | 11.52 | -3.33 | 0.0009 |
| 7 | prot=a4 | Legumin B | 2.60 | 34.78 | 12.59 | 2.73 | 0.0063 |
| | prot=b39 | Glucan Phosphorylase (faba) | 20.00 | 4.35 | 0.20 | 1.99 | 0.0470 |
| 8 | prot=a1 | Vicilin | 3.56 | 56.14 | 36.70 | 2.99 | 0.0027 |
| | prot=a3 | LMW Glutenin (wheat) | 0.71 | 3.51 | 11.52 | -2.06 | 0.0390 |
| 9 | prot=a1 | Vicilin | 8.91 | 55.94 | 36.70 | 4.80 | |
| | prot=a9 | p54 | 15.75 | 13.99 | 5.19 | 4.14 | |

| | | | | | | | |
|---|---|---|---|---|---|---|---|
| | prot=b14 | Lipoxygenase (pea) | 14.47 | 769 | 3.11 | 2.80 | |
| | prot=b24 | Albumin (pea) | 17.24 | 3.50 | 1.19 | 2.17 | |
| | prot=a7 | Legumin A2 | 2.88 | 4.20 | 8.50 | -2.00 | |
| | prot=b12 | Gamma Gliadin (wheat) | 000 | 000 | 2.82 | -2.44 | |
| | prot=a8 | Alpha-Gliadin (wheat) | 0.93 | 0.70 | 437 | -2.50 | |
| | prot=b15 | HMW Glutenin (wheat) | 0.00 | 0.00 | 2.98 | -2.53 | |
| | prot=a4 | Legumin B | 2.27 | 4.90 | 12.59 | -3.12 | |
| | prot=a3 | LMW Glutenin (wheat) | 0.00 | 0.00 | 11.52 | -5.67 | |
| 10 | prot=a7 | Legumin A2 | 12.50 | 16.05 | 8.50 | 3.24 | 0.0012 |
| | prot=b29 | Seed biotinylated protein of 65 kDa (pea) | 33.33 | 3.09 | 0.61 | 3.05 | 0.0023 |
| | prot=b15 | HMW Glutenin (wheat) | 1.37 | 0.62 | 2.98 | -2.00 | 0.0458 |
| | prot=a3 | LMW Glutenin (wheat) | 2.48 | 4.32 | 11.52 | -3.26 | 00011 |
| 11 | prot=a7 | Legumin A2 | 2.88 | 31.58 | 8.50 | 2.85 | 0.0043 |
| 12 | prot=b16 | Lectin | 13.33 | 8.60 | 2.45 | 3.10 | 0.0019 |
| | prot=b15 | HMW Glutenin (wheat) | 10.96 | 8.60 | 2.98 | 2.69 | 0.0071 |
| | prot=b39 | Glucan Phosphorylase (faba) | 40.00 | 2.15 | 0.20 | 2.46 | 0.0138 |
| 13 | prot=a1 | Vicilin | 2.00 | 64.29 | 36.70 | 2.93 | 0.0034 |
| 14 | prot=a3 | LMW Glutenin (wheat) | 21.63 | 31.61 | 11.52 | 7.86 | 0.0000 |
| | prot=b12 | Gamma Gliadin (wheat) | 27.54 | 9.84 | 2.82 | 4.95 | 0.0000 |
| | prot=b15 | HMW Glutenin (wheat) | 23.29 | 8.81 | 2.98 | 4.12 | 0.0000 |
| | prot=b25 | Alpha-Amylase Inhibitor, tetrameric, chain CM13 (wheat) | 30.77 | 2.07 | 0.53 | 2.38 | 0.0174 |
| | prot=b14 | Lipoxygenase (pea) | 15.79 | 6.22 | 3.11 | 2.33 | 0.0196 |
| | prot=b38 | Tonoplastic intrinsic protein (faba) | 50.00 | 1.04 | 0.16 | 2.11 | 0.0352 |
| | prot=b16 | Lectin | 0.00 | 0.00 | 2.45 | -2.71 | 0.0068 |
| | prot=a9 | p54 | 0.79 | 0.52 | 5.19 | -3.62 | 0.0003 |
| | prot=a1 | Vicilin | 2.78 | 12.95 | 36.70 | -7.64 | 0.0000 |
| 15 | prot=a8 | Alpha-Gliadin (wheat) | 40.19 | 11.11 | 4.37 | 6.20 | 0.0000 |
| | prot=a3 | LMW Glutenin (wheat) | 29.08 | 21.19 | 11.52 | 6.02 | 0.0000 |
| | prot=b15 | HMW Glutenin (wheat) | 36.99 | 6.98 | 2.98 | 4.46 | 0.0000 |
| | prot=b26 | Elongation factor | 53.85 | 1.81 | 0.53 | 3.10 | 0.0019 |
| | prot=b28 | Heat shock protein 70 (pea) | 50.00 | 1.81 | 0.57 | 2.93 | 0.0034 |
| | prot=b12 | Gamma Gliadin (wheat) | 27.54 | 4.91 | 2.82 | 2.51 | 0.0120 |
| | prot=b56 | Dimeric Alpha-Amylase Inhibitor (wheat) | 100.00 | 0.52 | 0.08 | 2.24 | 0.0250 |
| | prot=b55 | Starch Synthase (wheat) | 100.00 | 0.52 | 0.08 | 2.24 | 0.0250 |
| | prot=b43 | Polyubiquin | 100.00 | 0.52 | 0.08 | 2.24 | 0.0250 |
| | prot=b37 | Cu/Zn Superoxide Dismutase (pea) | 60.00 | 0.78 | 0.20 | 2.13 | 0.0333 |
| | prot=a9 | p54 | 8.66 | 2.84 | 5.19 | -2.38 | 0.0171 |
| | prot=a7 | Legumin A2 | 6.73 | 3.62 | 8.50 | -4.07 | 0.0000 |
| | prot=a4 | Legumin B | 7.14 | 5.68 | 12.59 | -4.81 | 0.0000 |
| | prot=a1 | Vicilin | 8.13 | 18.86 | 36.70 | -8.26 | 0.0000 |
| 16 | prot=a4 | Legumin B | 2.60 | 36.36 | 12.59 | 2.84 | 0.0045 |
| 17 | prot=a3 | LMW glutenin (wheat) | 38.30 | 24.16 | 11.52 | 8.49 | 0.0000 |
| | prot=a8 | Alpha-Gliadin (wheat) | 28.97 | 6.94 | 4.37 | 2.77 | 0.0057 |

| | | | | | | | |
|---|---|---|---|---|---|---|---|
| | prot=b34 | Alpha-Amylase Inhibitor. tetrameric, chain CM16 (wheat) | 100.00 | 0.67 | 0.12 | 2.74 | 0.0061 |
| | prot=b12 | Gamma Gliadin (wheat) | 30.43 | 4.70 | 2.82 | 2.49 | 0.0129 |
| | prot=b59 | COX 3 mitochondrion (wheat) | 100.00 | 0.45 | 0.08 | 2.13 | 0.0333 |
| | prot=b44 | CM2 (protein (wheat) | 100.00 | 0.45 | 0.08 | 2.13 | 0.0333 |
| | prot=a9 | p54 | 10.24 | 2.91 | 5.19 | -2.52 | 0.0117 |
| | prot=a4 | Legumin B | 12.99 | 8.95 | 12.59 | -2.64 | 0.0083 |
| | prot=a1 | Vicilin | 13.25 | 26.62 | 36.70 | -4.97 | 0.0000 |
| 18 | prot=a8 | Alpha-Gliadin (wheat) | 3.74 | 16.67 | 4.37 | 2.29 | 0.0219 |

The parent protein of a unique vegetable source is referred to with name of the protein in parentheses with Cla/Mod[a]: occurrence of the modality category in the cluster divided by its occurrence in the entire dataset; Mod/Cla[b]: proportion of the modality category within the cluster; Global[c] : global proportion of this modality category within the entire dataset.
