# Supplementary material for: Effect of protein aggregation in wheat-legume mixed pasta diets on their in vitro digestion kinetics in comparison to “rapid” and “slow” animal proteins
Source: PLoS One. 2020 May 4;15(5):e0232425. doi: 10.1371/journal.pone.0232425 (PMC7197814; doi:10.1371/journal.pone.0232425)
Supplement: S3 Table — (PDF) [file pone.0232425.s005.pdf]

S2 Table B. Relationship between the 18 clusters and some quantitative modalities (some amino-acid contents and digestive times) analyzed with the chi-square test ($p < 0.05$).

Cluster	Modality	Mean ± sd in category	Mean ± sd in global	v.test	p.value
1	G60-L	7.70 ± 0.63	3.54 ± 3.71	9	0.0000
	i120-P	0.00 ± 0.00	0.78 ± 2.23	-2.79	0.0052
	i120-F	0.00 ± 0.00	0.83 ± 2.36	-2.84	0.0045
	i120-L	0.00 ± 0.00	0.82 ± 2.31	-2.87	0.0041
	T0-F	0.00 ± 0.00	0.85 ± 2.28	-3.01	0.0026
	glutamine	0.75 ± 1.01	2.13 ± 3.20	-3.48	0.0005
	G60-F	0.00 ± 0.00	4.15 ± 3.82	-8.73	0.0000
	G60-P	0.00 ± 0.00	4.60 ± 3.68	-10.06	0.0000
2	G60-P	7.49 ± 0.62	4.60 ± 3.68	14.98	0.0000
	T0-L	0.00 ± 0.00	0.07 ± 0.66	-2.06	0.0390
	T0-P	0.00 ± 0.00	0.15 ± 0.96	-2.98	0.0029
	i120-P	0.00 ± 0.00	0.78 ± 2.23	-6.63	0.0000
	i120-F	0.00 ± 0.00	0.83 ± 2.36	-6.74	0.0000
	i120-L	0.00 ± 0.00	0.82 ± 2.31	-6.81	0.0000
	glutamine	0.95 ± 1.18	2.13 ± 3.20	-7.04	0.0000
	T0-F	0.00 ± 0.00	0.85 ± 2.28	-7.14	0.0000
	G60-L	0.00 ± 0.00	3.54 ± 3.71	-18.19	0.0000
	G60-F	0.00 ± 0.00	4.15 ± 3.82	-20.72	0.0000
3	T0-F	6.88 ± 0.57	0.85 ± 2.28	40.5	0.0000
	leucine	1.33 ± 0.95	0.90 ± 0.86	7.71	0.0000
	T0-P	0.00 ± 0.00	0.15 ± 0.96	-2.39	0.0170
	glutamine	1.51 ± 2.11	2.13 ± 3.20	-2.96	0.0031
	i120-P	0.00 ± 0.00	0.78 ± 2.23	-5.32	0.0000
	i120-F	0.00 ± 0.00	0.83 ± 2.36	-5.41	0.0000
	i120-L	0.00 ± 0.00	0.82 ± 2.31	-5.46	0.0000
	G60-L	0.00 ± 0.00	3.54 ± 3.71	-14.59	0.0000
	G60-F	0.00 ± 0.00	4.15 ± 3.82	-16.63	0.0000
	G60-P	0.00 ± 0.00	4.60 ± 3.68	-19.16	0.0000
4	i120-L	7.04 ± 0.75	0.82 ± 2.31	12.65	0.0000
	G60-L	0.00 ± 0.00	3.54 ± 3.71	-4.49	0.0000
	G60-F	0.00 ± 0.00	4.15 ± 3.82	-5.12	0.0000
	G60-P	0.00 ± 0.00	4.60 ± 3.68	-5.9	0.0000
5	G60-F	7.79 ± 0.71	4.15 ± 3.82	13.11	0.0000
	T0-P	0.00 ± 0.00	0.15 ± 0.96	-2.15	0.0320
	glutamine	1.57 ± 2.51	2.13 ± 3.20	-2.4	0.0160
	i120-P	0.00 ± 0.00	0.78 ± 2.23	-4.78	0.0000
	i120-F	0.00 ± 0.00	0.83 ± 2.36	-4.86	0.0000

	i120-L	0.00	±	0.00	0.82	±	2.31	-4.91	0.0000
	T0-F	0.00	±	0.00	0.85	±	2.28	-5.15	0.0000
	G60-L	0.00	±	0.00	3.54	±	3.71	-13.12	0.0000
	G60-P	0.00	±	0.00	4.60	±	3.68	-17.23	0.0000
6	T0-P	4.68	±	2.74	0.15	±	0.96	36.05	0.0000
	T0-L	2.39	±	3.07	0.07	±	0.66	26.82	0.0000
	T0-F	3.72	±	3.42	0.85	±	2.28	9.61	0.0000
	i120-F	0.10	±	0.77	0.83	±	2.36	-2.36	0.0180
	leucine	0.63	±	0.85	0.90	±	0.86	-2.41	0.0160
	i120-P	0.00	±	0.00	0.78	±	2.23	-2.65	0.0080
	i120-L	0.00	±	0.00	0.82	±	2.31	-2.72	0.0064
	G60-L	0.26	±	1.36	3.54	±	3.71	-6.75	0.0000
	G60-F	0.25	±	1.32	4.15	±	3.82	-7.79	0.0000
	G60-P	0.40	±	1.71	4.60	±	3.68	-8.73	0.0000
7	i120-F	6.89	±	0.71	0.83	±	2.36	12.37	0.0000
	G60-L	0.00	±	0.00	3.54	±	3.71	-4.59	0.0000
	G60-F	0.00	±	0.00	4.15	±	3.82	-5.23	0.0000
	G60-P	0.00	±	0.00	4.60	±	3.68	-6.03	0.0000
8	i120-P	7.18	±	0.87	0.78	±	2.23	21.92	0.0000
	i120-L	3.40	±	3.63	0.82	±	2.31	8.51	0.0000
	i120-F	2.23	±	3.46	0.83	±	2.36	4.52	0.0000
	T0-P	0.41	±	1.50	0.15	±	0.96	2.08	0.0380
	leucine	0.60	±	0.77	0.90	±	0.86	-2.72	0.0065
	glutamine	0.91	±	1.70	2.13	±	3.20	-2.91	0.0036
	G60-L	0.00	±	0.00	3.54	±	3.71	-7.28	0.0000
	G60-F	0.00	±	0.00	4.15	±	3.82	-8.29	0.0000
	G60-P	0.00	±	0.00	4.60	±	3.68	-9.56	0.0000
9	G60-L	7.09	±	0.78	3.54	±	3.71	11.79	0.0000
	G60-P	7.51	±	0.70	4.60	±	3.68	9.75	0.0000
	i120-P	0.00	±	0.00	0.78	±	2.23	-4.28	0.0000
	i120-F	0.00	±	0.00	0.83	±	2.36	-4.35	0.0000
	i120-L	0.00	±	0.00	0.82	±	2.31	-4.4	0.0000
	T0-F	0.00	±	0.00	0.85	±	2.28	-4.61	0.0000
	glutamine	0.79	±	1.15	2.13	±	3.20	-5.17	0.0000
	G60-F	0.00	±	0.00	4.15	±	3.82	-13.38	0.0000
10	G60-F	7.19	±	0.89	4.15	±	3.82	10.5	0.0000
	G60-P	7.44	±	0.88	4.60	±	3.68	10.17	0.0000
	T0-P	0.00	±	0.00	0.15	±	0.96	-2.06	0.0400
	i120-P	0.00	±	0.00	0.78	±	2.23	-4.57	0.0000
	i120-F	0.00	±	0.00	0.83	±	2.36	-4.65	0.0000
	i120-L	0.00	±	0.00	0.82	±	2.31	-4.7	0.0000
	T0-F	0.00	±	0.00	0.85	±	2.28	-4.93	0.0000
	G60-L	0.00	±	0.00	3.54	±	3.71	-12.55	0.0000

11	i120-F	7.36	±	0.88	0.83	±	2.36	12.1	0.0000
	i120-L	7.18	±	0.84	0.82	±	2.31	12.02	0.0000
	G60-L	0.00	±	0.00	3.54	±	3.71	-4.17	0.0000
	G60-F	0.00	±	0.00	4.15	±	3.82	-4.75	0.0000
	G60-P	0.00	±	0.00	4.60	±	3.68	-5.48	0.0000
12	G60-L	7.08	±	0.89	3.54	±	3.71	9.38	0.0000
	G60-F	7.42	±	0.83	4.15	±	3.82	8.41	0.0000
	leucine	0.67	±	0.77	0.90	±	0.86	-2.69	0.0070
	i120-P	0.00	±	0.00	0.78	±	2.23	-3.41	0.0006
	i120-F	0.00	±	0.00	0.83	±	2.36	-3.47	0.0005
	i120-L	0.00	±	0.00	0.82	±	2.31	-3.51	0.0005
	T0-F	0.00	±	0.00	0.85	±	2.28	-3.68	0.0002
	G60-P	0.00	±	0.00	4.60	±	3.68	-12.3	0.0000
13	T0-F	6.94	±	0.63	0.85	±	2.28	14.21	0.0000
	G60-F	7.79	±	0.82	4.15	±	3.82	5.07	0.0000
	G60-L	0.99	±	2.43	3.54	±	3.71	-3.66	0.0003
	G60-P	1.70	±	2.96	4.60	±	3.68	-4.2	0.0000
14	i120-F	7.53	±	0.68	0.83	±	2.36	41.06	0.0000
	i120-P	7.09	±	0.80	0.78	±	2.23	40.93	0.0000
	i120-L	7.26	±	0.68	0.82	±	2.31	40.25	0.0000
	glutamine	3.17	±	3.16	2.13	±	3.20	4.69	0.0000
	T0-P	0.00	±	0.00	0.15	±	0.96	-2.26	0.0240
	leucine	0.71	±	0.73	0.90	±	0.86	-3.24	0.0012
	T0-F	0.00	±	0.00	0.85	±	2.28	-5.42	0.0000
	G60-L	0.00	±	0.00	3.54	±	3.71	-13.8	0.0000
	G60-F	0.00	±	0.00	4.15	±	3.82	-15.72	0.0000
	G60-P	0.00	±	0.00	4.60	±	3.68	-18.11	0.0000
15	G60-L	6.79	±	0.60	3.54	±	3.71	18.77	0.0000
	G60-F	7.14	±	0.59	4.15	±	3.82	16.79	0.0000
	G60-P	6.91	±	0.66	4.60	±	3.68	13.45	0.0000
	glutamine	3.57	±	4.21	2.13	±	3.20	9.62	0.0000
	T0-L	0.00	±	0.00	0.07	±	0.66	-2.32	0.0200
	T0-P	0.00	±	0.00	0.15	±	0.96	-3.35	0.0008
	i120-P	0.00	±	0.00	0.78	±	2.23	-7.45	0.0000
	i120-F	0.00	±	0.00	0.83	±	2.36	-7.57	0.0000
	i120-L	0.00	±	0.00	0.82	±	2.31	-7.65	0.0000
	T0-F	0.00	±	0.00	0.85	±	2.28	-8.02	0.0000
16	i120-F	7.02	±	1.69	0.83	±	2.36	12.35	0.0000
	i120-L	6.04	±	2.93	0.82	±	2.31	10.63	0.0000
	i120-P	5.46	±	3.07	0.78	±	2.23	9.88	0.0000
	G60-L	6.41	±	3.13	3.54	±	3.71	3.64	0.0003
	G60-F	6.98	±	2.88	4.15	±	3.82	3.49	0.0005
	G60-P	6.79	±	2.83	4.60	±	3.68	2.81	0.0050

	glutamine	0.77	±	1.78	2.13	±	3.20	-2	0.0450
17	G60-L	7.89	±	0.54	3.54	±	3.71	27.41	0.0000
	G60-F	8.02	±	0.55	4.15	±	3.82	23.7	0.0000
	G60-P	7.89	±	0.53	4.60	±	3.68	20.92	0.0000
	glutamine	2.78	±	3.74	2.13	±	3.20	4.77	0.0000
	T0-L	0.00	±	0.00	0.07	±	0.66	-2.53	0.0110
	T0-P	0.03	±	0.38	0.15	±	0.96	-3.04	0.0024
	i120-P	0.00	±	0.00	0.78	±	2.23	-8.12	0.0000
	i120-F	0.00	±	0.00	0.83	±	2.36	-8.26	0.0000
	i120-L	0.00	±	0.00	0.82	±	2.31	-8.34	0.0000
	T0-F	0.00	±	0.00	0.85	±	2.28	-8.75	0.0000
18	T0-F	7.21	±	0.65	0.85	±	2.28	13.73	0.0000
	T0-P	2.72	±	3.25	0.15	±	0.96	13.17	0.0000
	T0-L	0.91	±	2.06	0.07	±	0.66	6.29	0.0000
	G60-F	7.98	±	0.84	4.15	±	3.82	4.93	0.0000
	G60-L	6.83	±	2.20	3.54	±	3.71	4.37	0.0000
	G60-P	7.79	±	0.89	4.60	±	3.68	4.26	0.0000
	leucine	1.42	±	1.11	0.90	±	0.86	2.97	0.0030
