# Supplementary figures and images for: Effect of protein aggregation in wheat-legume mixed pasta diets on their in vitro digestion kinetics in comparison to “rapid” and “slow” animal proteins
Source: PLoS One. 2020 May 4;15(5):e0232425. doi: 10.1371/journal.pone.0232425 (PMC7197814; doi:10.1371/journal.pone.0232425)

Fig 3A

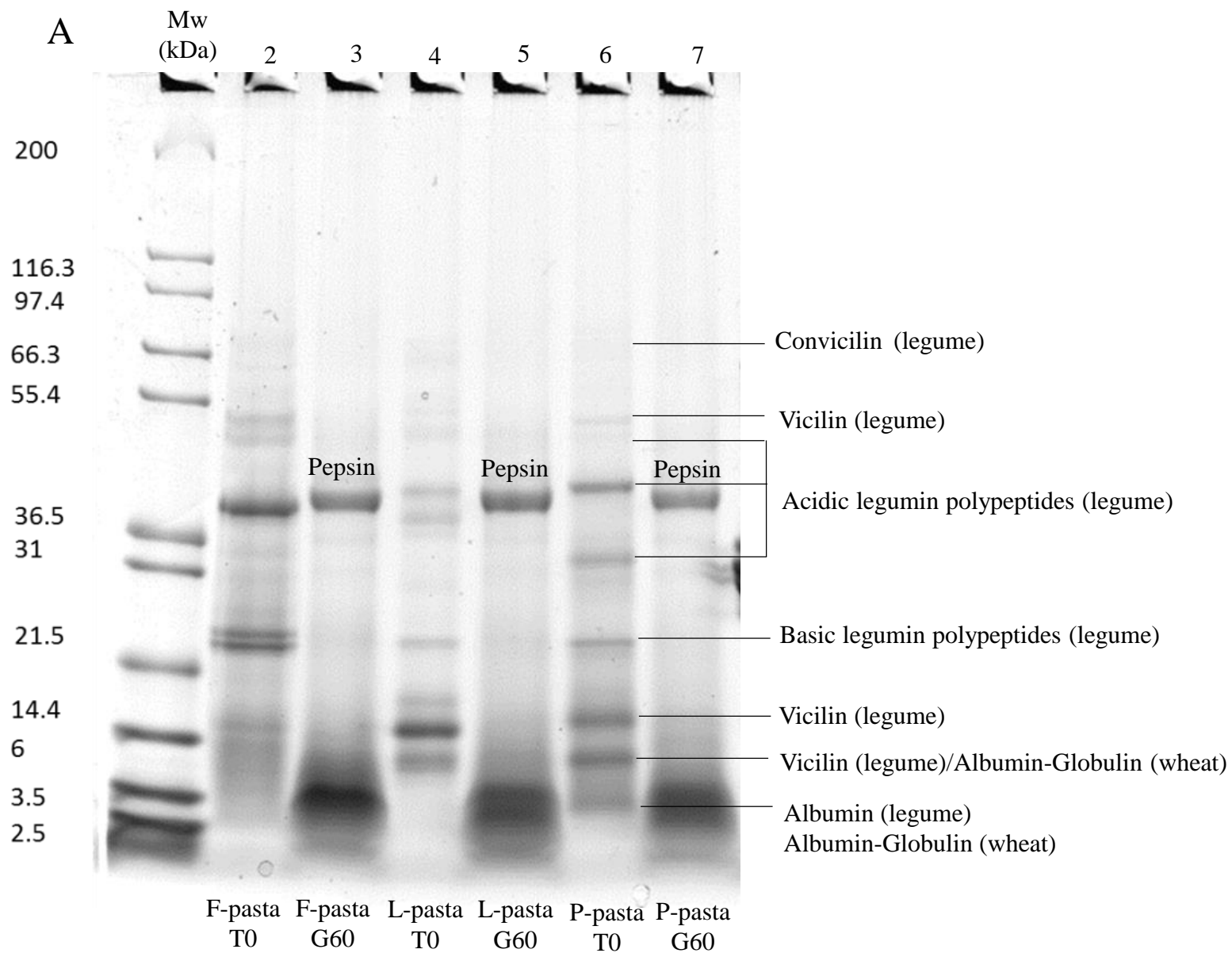

Fig 3B

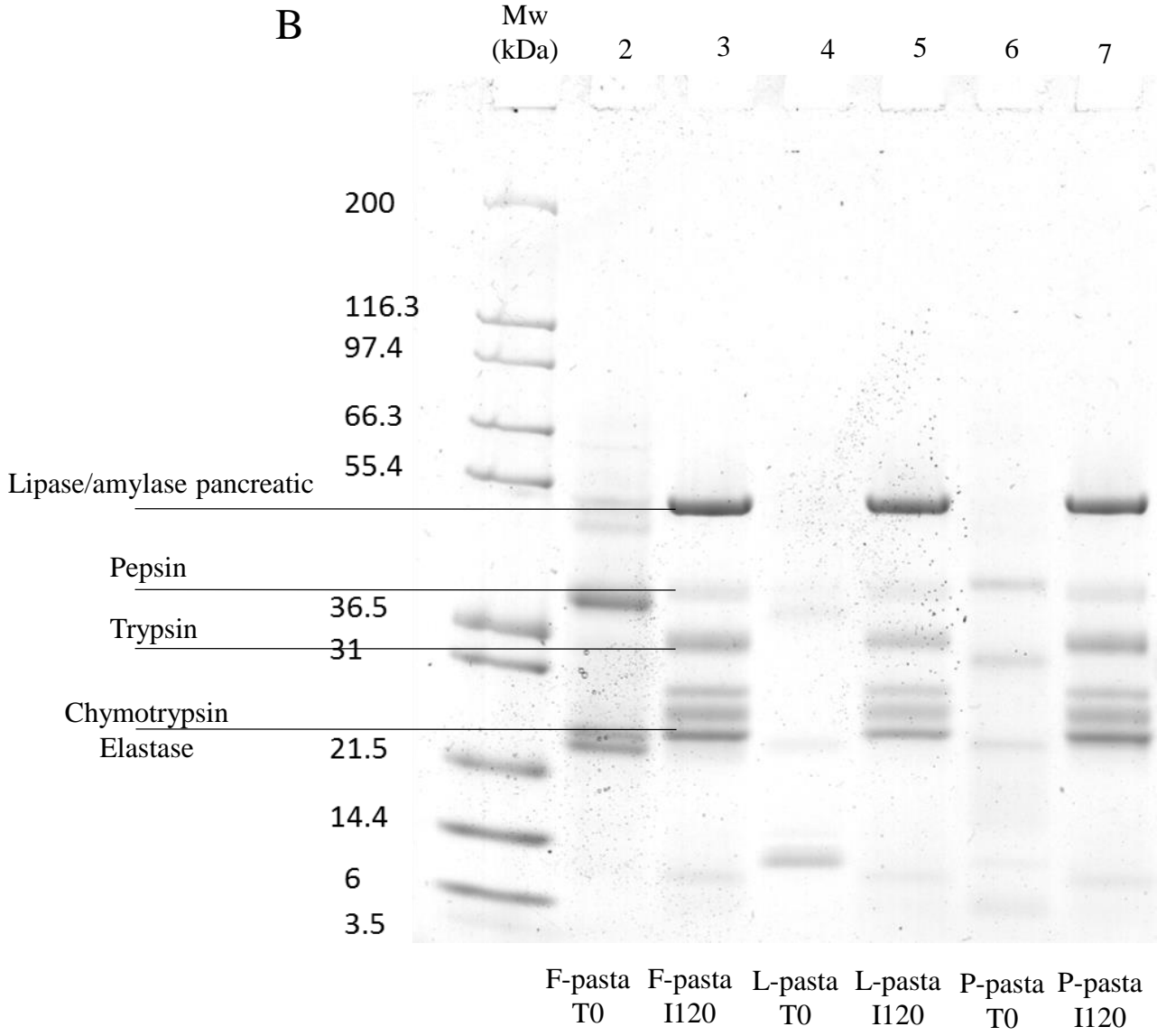

Fig 3C

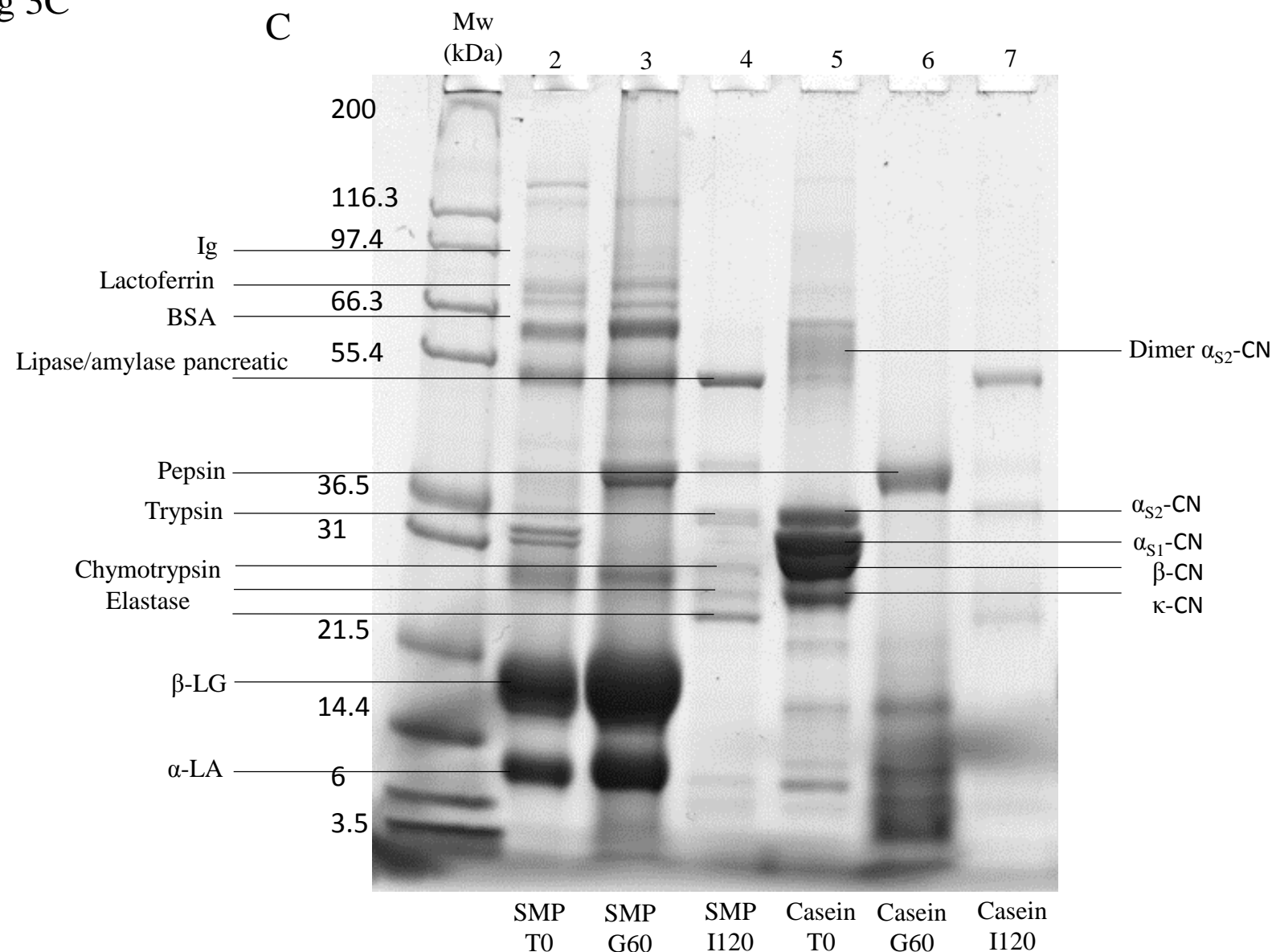

S1 Fig

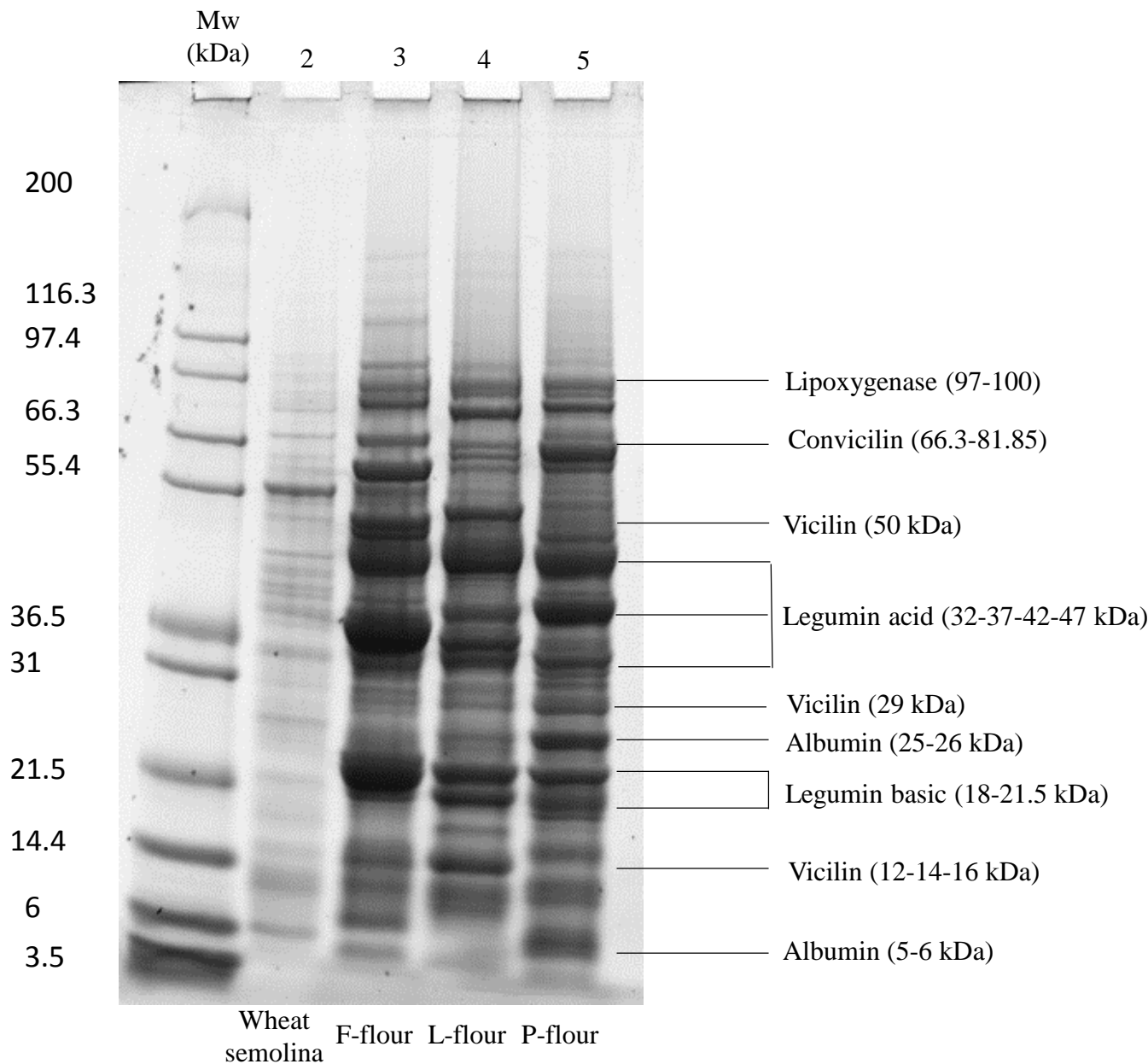

Supplement: S1 Raw image — (PDF) [file pone.0232425.s006.pdf]
